# Association between triglyceride glucose-body mass index and all-cause mortality in critically ill patients with atherosclerotic cardiovascular diseases: a retrospective cohort study using the MIMIC-IV database

**DOI:** 10.1186/s12872-026-06022-1

**Published:** 2026-05-29

**Authors:** Yaxin Liu, Chenxi Fu, Fengao Li, Hao Wang, Mei Zhu

**Affiliations:** 1 Department of Endocrinology and Metabolism, Tianjin Medical University General Hospital, Tianjin, 300052 China; 2 Department of General Surgery, Tianjin Medical University General Hospital, TianJin, 300052 China

**Keywords:** Atherosclerotic cardiovascular disease, Insulin resistance, TyG-BMI index, Intensive care, All-cause mortality

## Abstract

**Background:**

Atherosclerotic cardiovascular disease (ASCVD) remains the leading cause of cardiovascular mortality worldwide, with insulin resistance recognized as a key pathophysiological driver. While the triglyceride glucose-body mass index (TyG-BMI) has emerged as a promising insulin resistance marker, its prognostic value for ASCVD mortality outcomes requires further elucidation.

**Methods:**

We conducted a retrospective cohort study of 1,637 critically ill ASCVD patients (median age 68.3 years) from the MIMIC-IV database. The primary endpoints were 30-day and 1-year mortality, with secondary assessments of 90-day and 180-day mortality. Comprehensive statistical analyses included multivariable Cox regression, restricted cubic splines (RCSs) for nonlinearity assessment, and Kaplan-Meier survival analysis.

**Results:**

After full adjustment for potential confounders, elevated TyG-BMI showed significant inverse associations with both 30-day (HR 0.11, 95% CI 0.06–0.19) and 1-year mortality (HR 0.13, 95% CI 0.08–0.20). RCS analysis revealed a characteristic L-shaped dose-response relationship (nonlinear *P* < 0.001), while Kaplan-Meier curves demonstrated progressively improved survival across TyG-BMI quartiles (log-rank *P* < 0.001).

**Conclusion:**

This study revealed that the TyG-BMI is an independent predictor of mortality in critically ill ASCVD patients, with a distinct nonlinear protective effect. These findings support the potential clinical utility of the TyG-BMI for risk stratification in this high-risk population.

**Supplementary Information:**

The online version contains supplementary material available at 10.1186/s12872-026-06022-1.

## Introduction

 Cardiovascular diseases (CVDs) pose a significant health burden globally, representing approximately one in three deaths worldwide [1, 2]. Among CVDs, atherosclerotic cardiovascular disease (ASCVD)—primarily manifested as cardiac and cerebral ischemic events—has garnered significant clinical attention because of its distinct pathological and epidemiological characteristics [3]. As a clinically distinct disease entity, ASCVD shares common pathogenic mechanisms, modifiable risk factors, and preventive strategies [4]. Notably, ASCVD—particularly coronary artery disease—has been unequivocally identified as a primary driver of cardiovascular mortality worldwide [5]. Despite substantial advances in pharmacotherapy, revascularization techniques, and medical device innovation, ASCVD patients continue to exhibit considerable residual risk [6]. This highlights the critical necessity for developing innovative biomarkers to enhance risk assessment and improve treatment strategies.

The progression of ASCVD is significantly influenced by insulin resistance (IR), which occurs through several pathological processes such as sustained mild inflammation, vascular endothelial abnormalities, and disrupted metabolic homeostasis [7–9]. While recognized as the most accurate technique for measuring IR, the hyperinsulinaemic–euglycaemic clamp has limited clinical use owing to its complex methodology and significant resource requirements [10]. Unlike traditional methods, the triglyceride-glucose (TyG) index serves as an effective clinical biomarker for insulin resistance, demonstrating excellent prognostic capability in at-risk patients [11–13]. Emerging research has indicated that the TyG-BMI, which incorporates measures of adiposity, offers a more complete evaluation of insulin resistance and demonstrates an enhanced ability to predict both metabolic dysfunction and cardiovascular events [14–16]. Nevertheless, the clinical prognostic value of the TyG-BMI in ASCVD patients has not been established, highlighting the need for additional research.

In this study, the association of the TyG-BMI with mortality outcomes in critically ill ASCVD patients was evaluated through analysis of the MIMIC-IV database, with additional assessment of its supplementary predictive capacity. By clarifying this association, our research aims to support data-driven approaches for tailored risk assessment and individualized care strategies, thereby enhancing patient outcomes and alleviating health care system pressures. 

## Materials and methods

### Data source and study population

This analysis used anonymized ICU patient information from the Medical Information Mart for Intensive Care IV (MIMIC-IV version 3.1), an open-access database maintained by Beth Israel Deaconess Medical Center, a Harvard Medical School affiliate institution. The research team completed all the required ethical certifications (CITI Program certification number: 72856396), including training in data use agreements and conflict-of-interest management, to ensure compliance with database access requirements. The research cohort was established by applying the following exclusion criteria: (1) Individuals younger than 18 years of age; (2) Cases with in-hospital mortality within 24 h of admission; (3) Missing ICU admission records or repeated ICU stays; (4) ICU length of stay < 24 h; and (5) Incomplete core parameter records (including body mass index, lipid profiles, and glucose levels).

### Calculation of the TyG-BMI index

In this study, all indicators used to calculate the TyG-BMI index were derived from the first measurements obtained within 24 h of ICU admission. The TyG-BMI index was calculated through a sequential three-step process: first, the TyG index was derived using the formula ln[fasting glucose (mg/dL) × fasting triglycerides (mg/dL)]/2; second, BMI was calculated as weight (kg) divided by height squared (m²); finally, the TyG-BMI index was obtained by multiplying the TyG index by the BMI value. This composite index integrates both metabolic and anthropometric parameters to provide a comprehensive assessment of insulin resistance [16].

### Data collection

Patient characteristics at admission were retrieved from the MIMIC-IV database [17], including every available clinical metric documented within the initial 24-hour ICU period. The study employed ICD-9 and ICD-10 coding standards to verify clinical diagnoses, with exhaustive code listings presented in Table S1. In addition to the essential variables required for the TyG-BMI calculation, we collected the following data: (1) demographic characteristics (age, sex, and race) and hospitalization parameters (total hospital and ICU length of stay); (2) laboratory parameters and vital signs, such as the hemoglobin level; white blood cell count (WBC); platelet count, neutrophil count; aspartate aminotransferase (AST), alanine aminotransferase (ALT), albumin, total cholesterol (TC), low-density lipoprotein (LDL), high-density lipoprotein (HDL), blood urea nitrogen (BUN), creatinine, bicarbonate, sodium, potassium, and chloride levels; anion gap; prothrombin time (PT); partial thromboplastin time (PTT); international normalized ratio (INR); respiratory rate (RR); heart rate (HR); systolic blood pressure (SBP); diastolic blood pressure (DBP); and mean arterial pressure (MAP); (3) medication administration records, including antiplatelet agents, statins, insulin, β-blockers, ACE inhibitors/ARBs, diuretics, heparin, and warfarin; (4) preexisting comorbidities (heart failure, chronic obstructive pulmonary disease, renal disease, malignancy, hepatic disease, diabetes mellitus, hypertension, and atrial fibrillation); and (5) established critical illness assessment tools, such as the Sequential Organ Failure Assessment (SOFA), Acute Physiology Score III (APS III), Simplified Acute Physiology Score II (SAPS II), and Oxford Acute Severity of Illness Score (OASIS); and (6) critical interventions (mechanical ventilation and renal replacement therapy). All data extraction followed standardized protocols with quality assurance checks to ensure accuracy and completeness.

## Statistical analysis

Study subjects were categorized into four equal groups according to their TyG-BMI index quartile distribution (Q1: <297.3; Q2: 297.3-337.4; Q3: 337.4-433.1; Q4: >433.1), with baseline characteristics presented for each stratum. Continuous variables following normal distribution were presented as mean ± SD, while non-normally distributed data were expressed as median (IQR). For continuous variables, between-group differences were assessed with parametric t-tests or ANOVA when the data followed a normal distribution, and with nonparametric Mann-Whitney U tests when the data did not follow a normal distribution. Binary and categorical data were displayed as frequencies (percentages) and compared using χ² or Fisher’s exact tests depending on expected frequencies. To account for potential confounders, we conducted multivariable Cox regression analyses after confirming the absence of significant multicollinearity among covariates through variance inflation factor (VIF) assessment (all VIFs < 5, see Table S2). We developed four progressive statistical models: an unadjusted Model I; Model II with age, sex and race covariates; Model III incorporating additional physiological and biochemical adjustments (physiological and laboratory parameters including systolic/diastolic blood pressure, heart rate, respiratory rate, hemoglobin, white blood cell, albumin, BUN, creatinine, sodium, potassium and chloride); and Model IV (additionally adjusted for heart failure, COPD, malignancy, diabetes, hypertension, atrial fibrillation, antiplatelet agents, statins, insulin and diuretics) to systematically examine the unconfounded link between TyG-BMI and fatality risk. RCS modeling was utilized to examine potential nonlinear relationships between TyG-BMI and mortality outcomes. Subsequent stratification by TyG-BMI quartiles enabled detailed characterization of mortality risk gradients across different metabolic profiles. To assess the incremental predictive value of TyG-BMI, we incorporated it into established severity scoring systems (SOFA, SAPS II, APS III, OASIS) and evaluated model performance using Harrell’s C-statistic, with DeLong’s test comparing C-statistics between models with and without TyG-BMI. The incremental predictive value of continuous TyG-BMI was quantified through both IDI and NRI metrics when added to baseline models. Stratified analyses were performed to evaluate the consistency of the study findings across different demographic and clinical subgroups. The subgroup variables included age, sex, heart failure, atrial fibrillation, chronic obstructive pulmonary disease (COPD), hypertension, diabetes, and kidney disease. Statistical computations utilized R (v4.2) and SPSS (v25.0), applying a conventional two-sided significance threshold (*P* < 0.05).

## Results

### Study population and baseline characteristics

From an initial pool of 14,396 critically ill ASCVD patients in the MIMIC-IV database, our final analytical cohort comprised 1,637 individuals who satisfied all inclusion requirements (Figure S1). The baseline profiles showed no substantial variations between analyzed and non-analyzed cases (Table S3). The cohort demonstrated a median age of 68.3 years (IQR 59.7–77.7) with a male predominance (65.7%). Participants were categorized into quartiles according to their admission TyG-BMI values (Q1: <297.3; Q2: 297.3-337.4; Q3: 337.4-433.1; Q4: >433.1). Compared to the high TyG-BMI group, the low TyG-BMI group exhibited significantly lower levels of hemoglobin, white blood cell count, lymphocytes, neutrophils, albumin, TC, LDL, and bicarbonate (all *P* < 0.05), while demonstrating significantly higher levels of HDL, chloride, SAPS II, APS III, and OASIS scores (all *P* < 0.05). Furthermore, the low TyG-BMI group had significantly lower utilization rates of antiplatelet agents, statins, β-blockers, ACEI/ARBs, diuretics, and heparin (*P* < 0.05), lower prevalence of liver disease and diabetes mellitus, but higher prevalence of chronic obstructive pulmonary disease (COPD) and malignancies (*P* < 0.05). Importantly, low TyG-BMI group also showed significantly increased short-term and long-term mortality rates (30-d, 90-d, 180-d, and 1-year mortality; all *P* < 0.05) (Table 1).

**Table 1:** Baseline characteristics according to TyG-BMI quartiles

Variable	Overall(*N* = 1637)	Q1(*N* = 409)	Q2(*N* = 410)	Q3(*N* = 409)	Q4(*N* = 409)	*P* value
		< 297.3	297.3-337.4	337.4-433.1	> 433.1	
Age, years	68.3(59.7,77.7)	68.2(59.7,77.1)	68.5(60.5,78.6)	68.3(59.7,77.7)	68.2(59.1,77.5)	0.943
Gender, n (%)						0.743
Female	561(34.3)	147(35.9)	141(34.4)	141(33.7)	132(33.5)	
Male	1076(65.7)	262(64.1)	269(65.6)	268(66.3)	277(66.5)	
Ethnicity, n (%)						0.457
White	915(55.9)	238(57.2)	214(53.4)	231(55.7)	232(57.2)	
Black	122(7.4)	30(8.1)	33(7.6)	35(8.8)	24(5.4)	
Other	600(36.7)	141(34.7)	163(39.0)	143(35.5)	153(37.4)	
Hospital LOS, days	9.0(5.1,15.0)	8.9(4.9,14.8)	8.6(5.2,14.6)	9.2(5.1,15.8)	9.3(5.6,15.6)	0.784
ICU LOS, days	3.1(1.5,6.8)	3.2(1.5,7.2)	2.7(1.3,6.0)	3.0(1.6,6.5)	3.2(1.7,7.7)	0.335
Laboratory parameters						
Hemoglobin(g/dL)	11.7(10.0,13.2)	11.2(9.5,12.8)	11.6(10.1,13.3)	12.0(10.4,13.3)	11.8(10.1,13.2)	0.001
White blood cell(K/µL)	11.6(8.9,15.1)	11.6(8.6,15.0)	11.0(8.5,14.6)	11.6(9.3,14.9)	12.5(9.3,16.1)	0.001
Lymphocytes(K/µL)	1.4(0.9,2.1)	1.3(0.8,1.9)	1.4(0.8,2.1)	1.6(0.9,2.3)	1.6(1.0,2.3)	0.003
Neutrophils(K/µL)	9.0(6.2,12.7)	8.9(6.2,12.4)	8.6(5.8,12.0)	9.0(6.2,12.5)	9.7(6.9,14.2)	0.017
Platelets(K/µL)	194.0(149.0,247.0)	196.0(150.0,253.0)	189.0(146.5,242.5)	193.0(148.0,246.0)	196.5(154.0,250.0)	0.488
BUN(mg/dL)	18.0(13.5,27.5)	18.5(13.0,30.8)	18.0(13.0,25.0)	18.0(13.5,24.5)	19.0(14.0,29.0)	0.333
Creatinine(mg/dL)	1.0(0.8,1.4)	1.0(0.8,1.4)	1.0(0.8,1.4)	1.0(0.8,1.4)	1.0(0.8,1.5)	0.278
ALT(IU/L)	28.0(17.0,62.5)	25.0(14.0,57.0)	28.3(16.0,69.5)	29.0(18.0,58.3)	30.0(19.0,63.0)	0.194
AST(IU/L)	42.0(24.0,117.5)	39.5(23.0,109.8)	44.0(23.0,154.8)	39.8(23.5,116.5)	43.0(26.5,109.5)	0.762
Albumin(g/dL)	3.5(3.0,3.9)	3.4(2.8,3.8)	3.5(3.0,4.0)	3.6(3.2,4.0)	3.4(2.9,3.9)	0.019
TC(mg/dL)	158.0(126.0,194.0)	149.0(119.0,184.5)	157.0(125.0,191.0)	161.0(132.0,195.5)	168.0(135.5,202.5)	0.001
HDL(mg/dL)	44.0(35.0,55.0)	48.0(38.0,58.5)	46.0(36.0,56.5)	44.0(35.0,55.0)	39.0(32.0,48.0)	< 0.001
LDL(mg/dL)	84.0(60.0,115.0)	77.5(55.0,108.0)	84.0(61.0,113.0)	87.5(61.0,121.0)	89.5(62.0,120.0)	0.011
Bicarbonate(mEq/L)	22.5(20.5,24.0)	22.0(19.5,24.5)	22.5(20.0,24.0)	22.5(20.5,24.0)	23.0(21.0,24.5)	0.016
Sodium(mEq/L)	138.5(136.0,141.0)	139.0(136.5,141.5)	138.5(136.0,140.5)	138.5(137.0,140.5)	138.5(136.0,140.5)	0.076
Potassium(mEq/L)	4.3(4.0,4.6)	4.2(3.9,4.6)	4.2(4.0,4.6)	4.3(4.0,4.6)	4.3(4.0,4.7)	0.367
Chloride(mEq/L)	104.0(101.0,107.0)	105.0(101.0,107.0)	104.0(102.0,106.5)	104.5(101.5,107.0)	103.5(101.0,106.0)	0.001
Aniongap(mEq/L)	13.5(11.0,16.0)	14.0(11.5,16.0)	13.0(11.0,15.5)	13.5(11.0,16.0)	13.5(11.5,16.0)	0.136
INR	1.3(1.1,1.4)	1.3(1.1,1.5)	1.3(1.1,1.4)	1.2(1.1,1.4)	1.2(1.1,1.4)	0.406
PT(s)	13.5(12.2,15.4)	13.7(12.2,16.1)	13.5(12.4,15.5)	13.4(12.2,15.1)	13.4(12.1,15.1)	0.110
PTT(s)	32.7(27.8,49.3)	32.2(27.3,49.5)	34.3(28.4,51.6)	33.0(27.8,49.0)	31.9(27.8,46.8)	0.099
TyG-BMI	337.4(297.4,433.0)	273.6(254.5,286.4)	315.7(307.1,324.7)	406.5(394.5,421.5)	463.1(442.4,516.3)	< 0.001
Vital signs						
SBP, mmHg	116.3(107.2,130.2)	116.4(106.8,131.1)	115.7(106.8,128.3)	116.6(107.4,131.3)	117.5(108.1,131.2)	0.751
DBP, mmHg	63.8(57.2,73.1)	63.5(56.7,72.5)	63.6(57.2,71.2)	63.9(57.0,73.9)	65.1(58.4,75.1)	0.530
MBP, mmHg	79.4(73.7,88.5)	79.0(72.9,89.0)	78.7(73.4,86.7)	79.4(74.0,88.8)	80.4(74.5,89.9)	0.286
Heart Rate, bpm	80.3(71.9,89.9)	81.0(71.6,88.8)	79.2(71.2,89.2)	80.8(72.9,91.3)	79.9(72.0,91.4)	0.418
RR, times/min	18.9(17.0,21.5)	18.8(17.1,21.1)	18.8(16.9,21.3)	19.0(17.1,21.8)	19.2(17.2,21.6)	0.462
Disease severity scoring system						
SOFA	4.0(2.0,7.0)	4.0(2.0,7.0)	4.0(2.0,7.0)	4.0(2.0,7.0)	5.0(2.0,7.0)	0.157
SIRS	3.0(2.0,3.0)	3.0(2.0,3.0)	3.0(2.0,3.0)	3.0(2.0,3.0)	3.0(2.0,3.0)	0.132
SAPS II	35.0(27.0,44.0)	37.0(30.0,47.0)	35.0(28.0,44.0)	33.0(26.0,44.0)	34.0(27.0,41.0)	0.005
APS III	37.0(28.0,53.0)	41.0(30.0,56.0)	36.0(27.0,51.0)	34.0(26.0,52.0)	36.0(28.0,52.0)	< 0.001
OASIS	33.0(27.0,39.0)	34.0(29.0,40.0)	33.0(26.0,40.0)	31.0(26.0,39.0)	31.0(26.0,37.0)	< 0.001
Medications, n (%)						
Antiplatelet	1366(83.4)	319(78.0)	351(85.6)	354(86.6)	342(83.6)	0.005
Statin	1351(82.5)	309(75.6)	348(84.9)	353(86.3)	341(83.4)	< 0.001
Insulin	1264(77.2)	303(74.1)	317(77.3)	313(76.5)	331(80.9)	0.133
Beta blockers	1182(72.2)	269(65.8)	394(96.1)	315(77.0)	204(49.9)	0.003
ACEI/ARB	1414(86.4)	330(80.7)	360(87.8)	368(90.0)	356(87.0)	0.001
Diuretics	1089(66.5)	258(63.1)	261(63.7)	272(66.5)	298(72.9)	0.011
Heparin	1548(94.6)	374(91.4)	389(94.9)	391(95.6)	394(96.3)	0.011
Warfarin	265(16.2)	52(12.7)	71(17.3)	64(15.6)	78(19.1)	0.085
Comorbidities						
Congestive Heart Failure, n (%)	618(37.8)	167(40.8)	143(34.9)	152(37.2)	156(38.1)	0.365
COPD, n (%)	300(18.3)	73(17.8)	64(15.6)	65(15.9)	98(24.0)	0.006
Renal Disease, n (%)	326(19.9)	77(18.8)	80(19.5)	78(19.1)	91(22.2)	0.588
Malignant Cancer, n (%)	94(5.7)	36(8.8)	32(7.8)	15(3.7)	11(2.7)	< 0.001
Liver Disease, n (%)	88(5.4)	18(4.4)	18(4.4)	15(3.7)	37(9.0)	0.002
Diabetes, n (%)	586(35.8)	106(25.9)	130(31.7)	148(36.2)	202(49.4)	< 0.001
Hypertension, n (%)	755(46.1)	171(41.8)	196(47.8)	203(49.6)	185(45.2)	0.128
Atrial Fibrillation, n (%)	601(36.7)	161(39.4)	160(39.0)	135(33.0)	145(35.5)	0.181
Outcome, n (%)						
30-d mortality	315(19.2)	165(40.3)	79(19.3)	50(12.2)	21(5.1)	< 0.001
90-d mortality	366(22.4)	181(44.3)	97(23.7)	60(14.7)	28(6.8)	< 0.001
180-d mortality	408(24.9)	195(47.7)	110(26.8)	70(17.1)	33(8.1)	< 0.001
1 year mortality	453(27.7)	210(51.3)	127(31.0)	79(19.3)	37(9.0)	< 0.001
RRT, n (%)	171(10.4)	38(9.3)	44(10.7)	48(11.7)	41(10.0)	0.700
Ventilation, n (%)	1375(84.0)	339(82.9)	336(82.0)	346(84.6)	354(86.6)	0.290

*LOS* Length of Stay, *BUN* blood urea nitrogen, *ALT* alanine transaminase, *AST* aspartate aminotransferase, *TC* total cholesterol,* HDL* high-density lipoprotein, *LDL* low-density lipoprotein, *INR* international normalized ratio, *PT* prothrombin time, *PTT* partial thromboplastin time, *TyG-BMI* triglyceride glucose-body mass index, *SBP* systolic blood pressure, *DBP* diastolic blood pressure, *MBP* mean blood pressure, *RR* respiratory rate, *SOFA* Sequential Organ Failure Assessment, *SIRS* Systemic Inflammatory Response Syndrome, *SAPS II* Simplified Acute Physiology Score II, *APS III* Acute Physiology Score III, *OASIS* Oxford Acute Severity of Illness Score, *COPD* chronic obstructive pulmonary disease

### Association between TyG-BMI index and all-cause mortality in ASCVD patients

The impact of TyG-BMI on survival was quantified using multivariate Cox regression analysis in a cohort of severely ill ASCVD cases (Table 2). For analytical purposes, subjects were grouped into four equal quartiles (Q1 to Q4) determined by their TyG-BMI measurements. In the fully adjusted Model IV, compared with Q1, the HR with 95% CI for 30-d mortality were 0.58 (0.41–0.83) for Q2, 0.39 (0.27–0.58) for Q3, and 0.11 (0.06–0.19) for Q4 (P-trend < 0.001). Similarly, the corresponding HR (95% CI) for 1-year mortality were 0.63 (0.47–0.85), 0.39 (0.28–0.55), and 0.13 (0.08–0.20), respectively (P-trend < 0.001), demonstrating a significant inverse relationship between TyG-BMI levels and mortality risk.

**Table 2:** HRs (95% CIs) for 30-d and 1 year mortality according to TyG-BMI quartiles

Variable	Model I		Model II		Model III		Model IV	
	HR (95% CI)	*P* value	HR (95% CI)	*P* value	HR (95% CI)	*P* value	HR (95% CI)	*P* value
30-d mortality								
TyG-BMI quantile								
Q1	Ref.		Ref.		Ref.		Ref.	
Q2	0.42(0.32,0.55)	< 0.001	0.43(0.33,0.56)	< 0.001	0.63(0.45,0.88)	0.007	0.58(0.41,0.83)	0.002
Q3	0.26(0.19,0.36)	< 0.001	0.26(0.19,0.36)	< 0.001	0.42(0.29,0.61)	< 0.001	0.39(0.27,0.58)	< 0.001
Q4	0.11(0.07,0.17)	< 0.001	0.11(0.07,0.17)	< 0.001	0.13(0.07,0.21)	< 0.001	0.11(0.06,0.19)	< 0.001
P for trend		< 0.001		< 0.001		< 0.001		< 0.001
1 year mortality								
TyG-BMI quantile								
Q1	Ref.		Ref.		Ref.		Ref.	
Q2	0.50(0.40,0.63)	< 0.001	0.51(0.41,0.63)	< 0.001	0.66(0.45,0.88)	0.005	0.63(0.47,0.85)	0.002
Q3	0.30(0.23,0.38)	< 0.001	0.29(0.23,0.38)	< 0.001	0.41(0.30,0.56)	< 0.001	0.39(0.28,0.55)	< 0.001
Q4	0.13(0.09,0.19)	< 0.001	0.13(0.09,0.19)	< 0.001	0.15(0.10,0.22)	< 0.001	0.13(0.08,0.20)	< 0.001
P for trend		< 0.001		< 0.001		< 0.001		< 0.001

Model I: no adjustment

Model II: adjusted for age, sex and race

Model III: further adjusted for SBP, DBP, heart rate, respiratory rate, hemoglobin, white blood cell, albumin, BUN, creatinine, sodium, potassium, chloride

Model IV: further adjusted for congestive heart failure, COPD, malignant cancer, diabetes, hypertension, atrial fibrillation, antiplatelet, statin, insulin, diuretics

This consistent trend was also observed in 90-d and 180-d mortality analyses (Table S4) and remained robust after excluding patients with missing baseline data, malignancies, or extreme TyG-BMI values (Tables S5-S7).

**Fig. 1:**
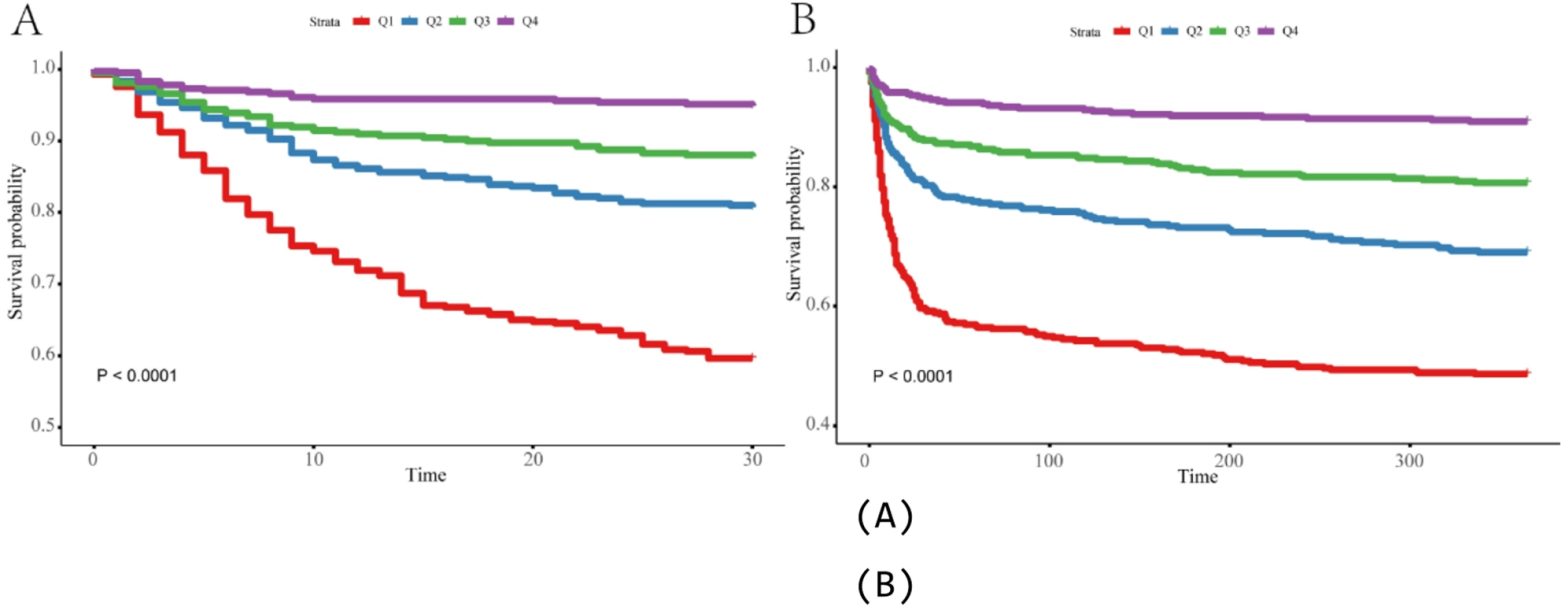
Kaplan-Meier survival curve for 30-d and 1 year mortality according to TyG-BMI. **A** Kaplan-Meier survival curve for 30-d mortality according to TyG-BMI; Log-rank test *P* < 0.0001. **B** Kaplan-Meier survival curve for 1 year mortality according to TyG-BMI; Log-rank test *P* < 0.0001. TyG-BMI: triglyceride glucose-body mass index

Kaplan-Meier survival curves stratified by TyG-BMI quartiles revealed progressively lower mortality risks with increasing TyG-BMI levels among critically ill ASCVD patients. Both 30-d and 1-year mortality rates showed gradual reductions with higher TyG-BMI levels (log-rank test, *P* < 0.001; Fig. 1). This protective trend was consistently demonstrated for 90-d and 180-d mortality as well (log-rank test, *P* < 0.001; Figure S2).

### Assessment of the nonlinear relationship between TyG-BMI index and all-cause mortality in ASCVD patients

**Fig. 2:**
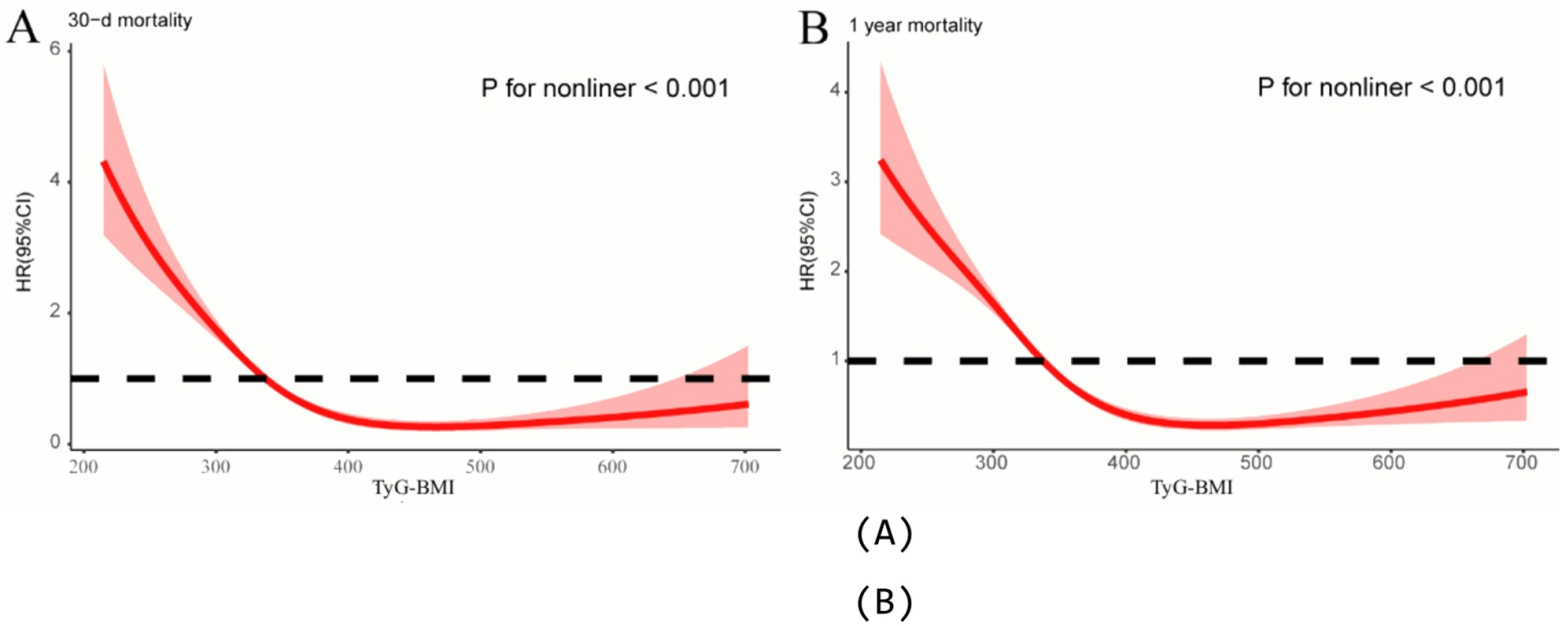
The potential non-linear relationship between TyG-BMI and 30-d/1 year mortality in patients with ASCVD was evaluated using a restricted cubic spline analysis. **A** A multiple-variable adjusted restricted cubic spline method was used to illustrate the relationship between TyG-BMI levels and 30-d mortality in ASCVD patients; **B** A multiple-variable adjusted restricted cubic spline method was used to illustrate the relationship between TyG-BMI levels and 1 year mortality in ASCVD patients. The solid line represents the estimated HR, and the red shaded area indicates the corresponding 95% CI. X-axis: TyG-BMI level; Y-axis: HR for mortality. HR: hazard ratio; CI: confidence interval; TyG-BMI: triglyceride glucose-body mass index

To examine nonlinear relationships between TyG-BMI and mortality risk in ASCVD patients, we employed restricted cubic spline (RCS) regression analysis. Results indicated a pronounced L-shaped dose-response relationship (P for nonlinearity < 0.001), with mortality risk sharply declining with rising TyG-BMI before leveling off, consistently observed at 30-d and 1-year endpoints (Fig. 2). The observed nonlinear relationship persisted consistently across 90-d and 180-d mortality evaluations (P for nonlinearity < 0.001; see Figure S3).

Bifurcated Cox models detected critical inflection points at TyG-BMI values of 449.3 (30-d mortality) and 448.8 (1-year mortality), indicating separate risk phases (Table 3). The prethreshold analysis revealed an inverse linear relationship, where every 1-unit TyG-BMI augmentation predicted significantly lower mortality (1.2% decrease for 30-d mortality: HR 0.988, 95% CI 0.986–0.989; 1.1% decrease for 1-year mortality: HR 0.989, 95% CI 0.987–0.990; both *P* < 0.001). Above the thresholds, mortality risk showed a slight but statistically significant increase (0.4% increase for 30-d mortality: HR 1.004, 95% CI 1.000-1.007, *P* = 0.038; 0.4% increase for 1-year mortality: HR 1.004, 95% CI 1.001–1.006, *P* = 0.012).

**Table 3:** Threshold analyses of TyG-BMI on outcome using two-piecewise regression models

	Adjusted HR (95%CI)	*P* value
30 day-mortality		
Fitting by the standard linear model	0.990(0.988,0.991)	< 0.001
Fitting by the two-piecewise linear model		
Inflection point	449.3	
TyG-BMI < 449.3	0.988(0.986,0.989)	< 0.001
TyG-BMI > 449.3	1.004(1.000,1.007)	0.038
* P* for Log-likelihood ratio		< 0.001
1 year mortality		
Fitting by the standard linear model	0.991(0.990–0.992)	< 0.001
Fitting by the two-piecewise linear model		
Inflection point	448.8	
TyG-BMI < 448.8	0.989(0.987–0.990)	< 0.001
TyG-BMI > 448.8	1.004(1.001–1.006)	0.012
* P* for Log-likelihood ratio		< 0.001

*TyG-BMI* triglyceride glucose-body mass index

For 90-d and 180-d mortality, the identified inflection point was 446.3. Below this threshold, mortality risk decreased significantly (HR 0.988 and 0.989, respectively; both *P* < 0.001), while above the threshold, risk showed marginal increases (HR 1.003 for both; *P* = 0.036 and *P* = 0.071, respectively) (Table S8).

### Predictive value and incremental effect of TyG-BMI index

**Fig. 3:**
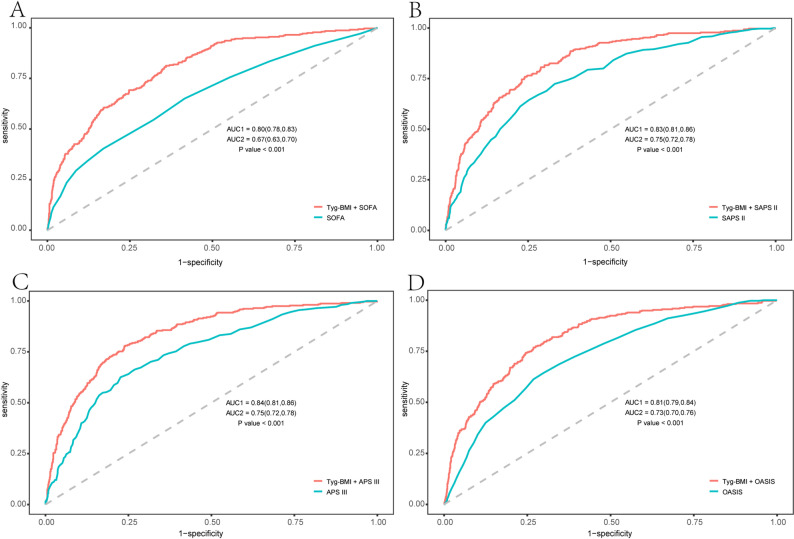
ROC curve analysis of the incremental effect of TyG-BMI on 30-d all-cause mortality. **A** SOFA + TyG-BMI; **B** SAPS II + TyG-BMI; **C** APS III + TyG-BMI; **D** OASIS + TyG-BMI. SOFA: Sequential Organ Failure Assessment; SAPS II: Simplified Acute Physiology Score II; APS III: Acute Physiology Score III; OASIS: Oxford Acute Severity of Illness Score; TyG-BMI: triglyceride glucose-body mass index

**Fig. 4:**
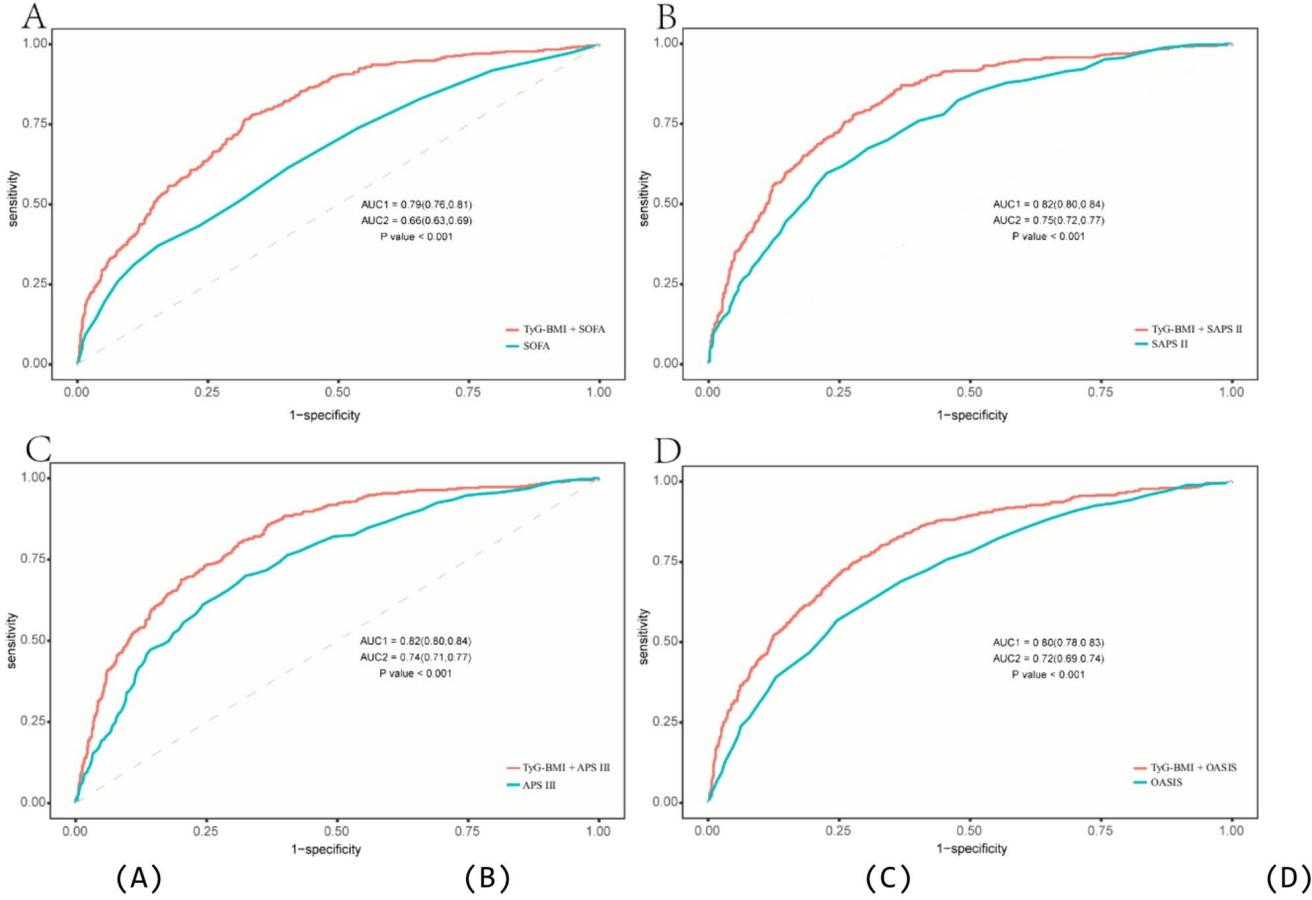
ROC curve analysis of the incremental effect of TyG-BMI on 1 year all-cause mortality. **A** SOFA + TyG-BMI; (**B**) SAPS II + TyG-BMI; (**C**) APS III + TyG-BMI; (**D**) OASIS + TyG-BMI. SOFA: Sequential Organ Failure Assessment; SAPS II: Simplified Acute Physiology Score II; APS III: Acute Physiology Score III; OASIS: Oxford Acute Severity of Illness Score; TyG-BMI: triglyceride glucose-body mass index

The incremental prognostic value of TyG-BMI was quantified through ROC curve comparisons, showing statistically significant enhancement in discrimination for both 30-d and 1 year mortality prediction when added to conventional risk assessment tools. Our findings revealed that incorporating TyG-BMI substantially improved discrimination in all predictive models for 30-d mortality: the AUC increased from 0.67 (95% CI: 0.63–0.70) to 0.80 (0.78–0.83) for SOFA, from 0.75 (0.72–0.78) to 0.83 (0.81–0.86) for SAPS II, from 0.75 (0.72–0.78) to 0.84 (0.81–0.86) for APS III, and from 0.73 (0.70–0.76) to 0.81 (0.79–0.84) for OASIS, with all improvements being statistically significant (*P* < 0.001; Fig. 3). Similar significant enhancements were observed for 1-year mortality prediction: AUC improved from 0.66 (0.63–0.69) to 0.79 (0.76–0.81) for SOFA, from 0.75 (0.72–0.77) to 0.82 (0.80–0.84) for SAPS II, from 0.74 (0.71–0.77) to 0.82 (0.80–0.84) for APS III, and from 0.72 (0.69–0.74) to 0.80 (0.78–0.83) for OASIS (*P* < 0.001; Fig. 4). Similar predictive enhancements were observed for both 90-d and 180-d mortality outcomes (Figures S4-S5).

Table 4 demonstrates that adding TyG-BMI notably enhanced prediction accuracy for mortality at 30 days and one year in all scoring models (all P-values for change in C-statistics < 0.001). For 30-d mortality prediction, the IDI indices were 0.16 for SOFA, 0.12 for SAPS II, 0.14 for APS III, and 0.15 for OASIS, with corresponding NRI values of 0.36, 0.32, 0.34, and 0.34, respectively. Regarding 1-year mortality prediction, the IDI values increased by 0.18 for SOFA, 0.14 for SAPS II, 0.16 for APS III, and 0.16 for OASIS, with corresponding NRI values of 0.35, 0.31, 0.34, and 0.33. The inclusion of TyG-BMI also yielded statistically significant improvements in discriminative performance for 90-d and 180-d mortality predictions (Table S9).

**Table 4:** Incremental value of TyG-BMI for 30-day and 1year mortality

	C-statistic^a^	C-statistic^b^	Δ C-statistic	*P*-value for Δ C	IDI (95% CI)	NRI (95% CI)
30 day-mortality						
SOFA + TyG-BMI vs. SOFA	0.64(0.59,0.69)	0.76(0.72,0.80)	0.12	< 0.001	0.16(0.02,0.21)	0.36(0.05,0.46)
SAPS II + TyG-BMI vs. SAPS II	0.73(0.69,0.78)	0.80(0.76,0.84)	0.07	< 0.001	0.12(0.03,0.17)	0.32(0.07.0.41)
APS III + TyG-BMI vs. APS III	0.69(0.64,0.74)	0.79(0.75,0.83)	0.10	< 0.001	0.14(0.03,0.17)	0.34(0.06,0.44)
OASIS + TyG-BMI vs. OASIS	0.70(0.65,0.74)	0.78(0.74,0.82)	0.08	< 0.001	0.15(0.02,0.17)	0.34(0.03,0.39)
1 year-mortality						
SOFA + TyG-BMI vs. SOFA	0.64(0.59,0.68)	0.75(0.72,0.78)	0.11	< 0.001	0.18(0.04,0.22)	0.35(0.08,0.41)
SAPS II + TyG-BMI vs. SAPS II	0.72(0.68,0.76)	0.79(0.76,0.82)	0.07	< 0.001	0.14(0.02,0.18)	0.31(0.06,0.37)
APS III + TyG-BMI vs. APS III	0.68(0.64,0.72)	0.78(0.74,0.81)	0.10	< 0.001	0.16(0.03,0.20)	0.34(0.06,0.44)
OASIS + TyG-BMI vs. OASIS	0.68(0.64,0.72)	0.76(0.73,0.80)	0.09	< 0.001	0.16(0.03,0.21)	0.33(0.06,0.39)

*SOFA* Sequential Organ Failure Assessment, *SAPS II* Simplified Acute Physiology Score II, *APS III* Acute Physiology Score III, *OASIS* Oxford Acute Severity of Illness Score, *FI-LAB* Frailty Index based on Laboratory, *TyG-BMI* triglyceride glucose-body mass index

### Subgroup analysis

**Fig. 5:**
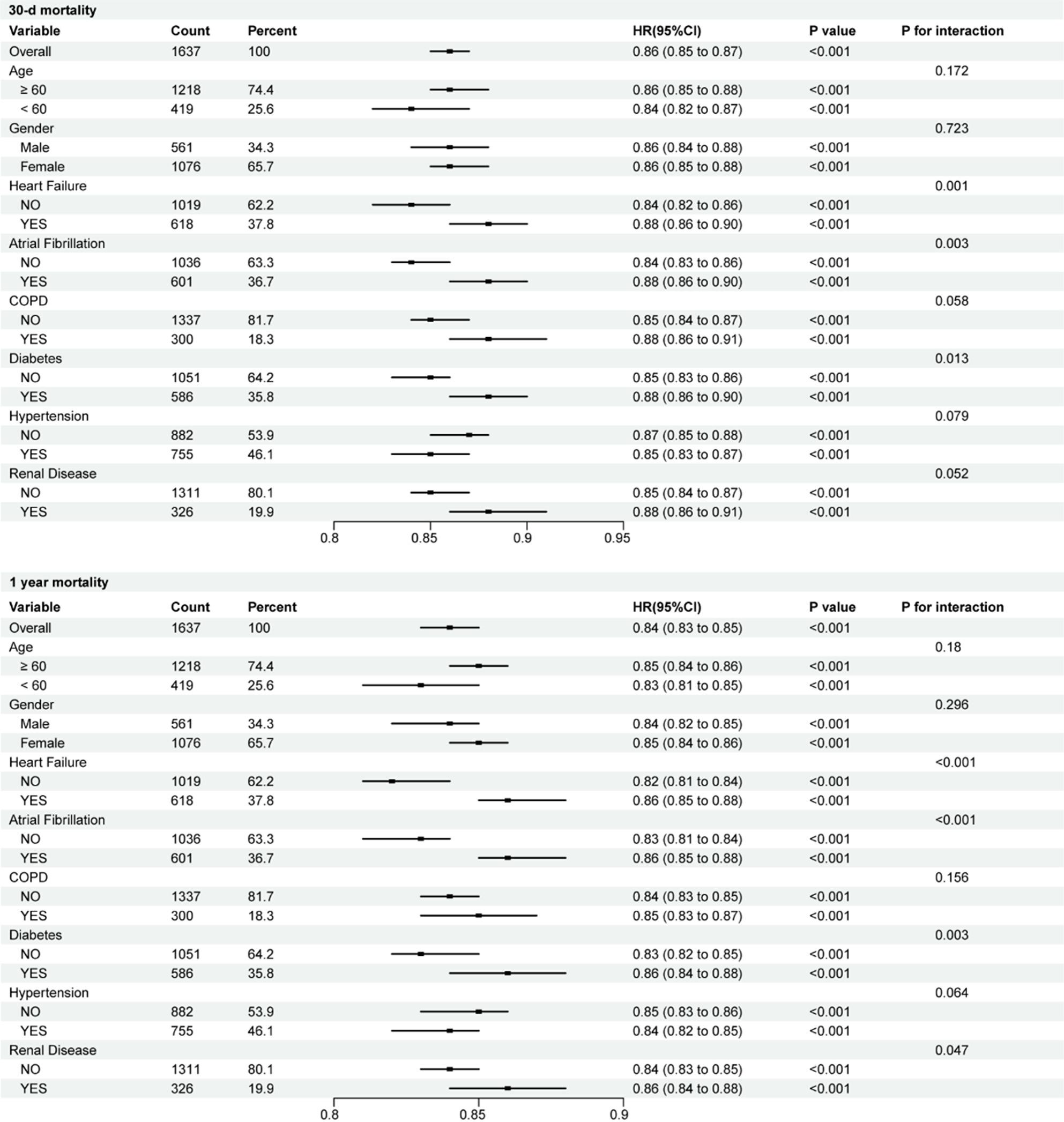
Subgroup analyses for the correlation of TyG-BMI with risk of 30-d and 1 year mortality in ASCVD patients. COPD: chronic obstructive pulmonary disease; HR: hazard ratio; CI: confidence interval

Comprehensive subgroup analyses were performed to evaluate the robustness of the TyG-BMI-mortality association across various clinical presentations in the ASCVD cohort (Fig. 5 and Figure S6). Stratification variables included age, sex, heart failure, atrial fibrillation, COPD, diabetes mellitus, hypertension, and renal disease. Analysis showed that TyG-BMI did not significantly interact with mortality rates (at 30-d, 90-d, 180-d, or 1 year) in subgroups stratified by age, gender, COPD, or hypertension (all interaction P-values > 0.05). Notably, the protective association of TyG-BMI was more pronounced in patients without comorbid heart failure, atrial fibrillation, or diabetes mellitus (all interaction P-values < 0.05 for all time points). Regarding renal disease, while no significant interaction was observed for 30-d and 90-d mortality (interaction P-values > 0.05), TyG-BMI showed a stronger association with reduced mortality risk at 180 days and 1 year in patients without renal disease (interaction P-values < 0.05).

## Discussion

Examination of 1,637 critical ASCVD patients from the MIMIC-IV database revealed an L-shaped TyG-BMI-mortality association that has not been previously described. The inverse relationship of the index with mortality outcomes at all time intervals, coupled with its independent predictive capacity for increased risk at lower values, suggests clinical relevance for patient assessment.

Substantial research has conclusively demonstrated the pivotal role of insulin resistance (IR) in both the development and advancement of ASCVD. Numerous population-based studies employing various IR evaluation methods have reported congruent results. Notably, the PURE study assessed 141,243 urban and rural residents aged 35 to 70 years across 22 countries at baseline. After a median follow-up of 13.2 years, the findings confirmed that a higher TyG index serves as a strong predictor of cardiovascular mortality, myocardial infarction, cerebrovascular events, and incident type 2 diabetes [18]. In the Chinese Kailuan cohort study, baseline measurements of fasting blood glucose levels, triglyceride levels, and BMI were taken from 95,342 participants without ASCVD. After up to 15 years of follow-up, both the standard TyG index and its BMI-adjusted versions—including those incorporating waist circumference and waist-to-height ratio—were significantly and positively correlated with the occurrence of ASCVD [19]. Multiple insulin resistance evaluation tools have shown robust cardiovascular prognostic capacity: A study focusing on community-dwelling older Chinese adults without baseline cardiovascular disease and with complete estimated glucose disposal rate (eGDR) data revealed that after a median follow-up of 106.5 months, lower eGDR levels were significantly associated with an increased risk of incident cardiovascular disease [20]; another study involving 2,515 U.S. residents with cardiovascular disease analysed their baseline metabolic score for insulin resistance (METS-IR) and, after a median follow-up of 91.4 months, reported that the lnMETS-IR exhibited a nonlinear U-shaped curve relationship with both all-cause and cardiovascular mortality [21]. Additionally, a study incorporating 6,755 adults aged 40 to 69 years from the Korean Epidemiology and Genomics Study revealed that elevated homeostatic model assessment for insulin resistance (HOMA-IR) levels had a positive dose-response association with cardiovascular mortality [22]. These comprehensive findings collectively underscore the pivotal role of IR in ASCVD development, progression, and outcome prediction across diverse populations and clinical contexts.

The observed inverse relationship between the TyG-BMI and mortality risk in our critically ill ASCVD cohort is notably different from prior findings in younger Northern Chinese subjects, where elevated TyG indices predicted increased mortality following premature ASCVD events [23]. Such a discrepancy suggests potential population-specific variations in the clinical significance of different insulin resistance indices. The TyG-BMI index, as an extended version of the conventional TyG index that incorporates BMI, may provide a more comprehensive reflection of insulin resistance status [24]. Current studies assessing the association between the TyG-BMI and cardiovascular prognosis have produced inconsistent results. Notably, Luo’s research team documented an important U-curve correlation, demonstrating elevated mortality at both extremes of TyG-BMI values during 90 days, 180 days, and 1 year of follow-up in critically ill patients with myocardial infarction [25]. In contrast to these findings, a large American cardiovascular cohort revealed that increased TyG-BMI values were independently predictive of lower all-cause mortality rates [14]. Additionally, investigations in older ambulatory diabetic patients demonstrated a dose-dependent relationship, with progressively higher TyG-BMI values corresponding to increased cardiovascular mortality risk [26]. These divergent observations underscore the potential context-dependent prognostic value of the TyG-BMI across different clinical settings and patient populations, warranting further investigation.

In light of traditional views that uniformly link obesity to cardiovascular risk, recent studies have reported an “obesity paradox”—a counterintuitive survival advantage among overweight or moderately obese individuals in certain subgroups, especially older adults and critically ill individuals [27]. Multiple clinical investigations have consistently shown that overweight and grade I obese CVD patients have more favorable short- and intermediate-term prognoses than their nonobese counterparts do [28, 29]. The study population had a median age of 68.3 years and consisted exclusively of critically ill patients. In this vulnerable geriatric population with critical illness, nutritional status emerges as a crucial determinant of mortality risk. Moderate adiposity may confer survival advantages through multiple mechanisms, including the attenuation of frailty progression and the prevention of cachexia development [30]. Although BMI serves as a commonly used yet relatively crude indicator for obesity assessment, lower BMI values may reflect underlying conditions, including sarcopenia, malnutrition, hypercatabolic states, reduced physical activity, or the presence of other comorbidities [31]. Furthermore, mild overweight status in elderly people may provide essential metabolic reserves that protect against adverse health outcomes including frailty syndrome, nutritional deficiencies, and osteoporosis [32]. Longitudinal studies have additionally identified declining BMI trajectories as sensitive biomarkers of deteriorating health status in aging populations, with such reductions demonstrating significant associations with elevated all-cause mortality risk [33–35].

Emerging evidence suggests that obesity may be associated with reduced mortality in critically ill patients, a phenomenon supported by several meta-analyses and potentially mediated through multiple pathophysiological mechanisms [36–38]. In particular, obese individuals possess greater energy reserves that may help sustain metabolic demands during the hypercatabolic state induced by critical illness [39]. Second, the chronic low-grade inflammatory state characteristic of obesity might induce “inflammatory tolerance” or “preconditioning” effects, thereby attenuating acute inflammatory injury [40]. Additionally, adipokines such as leptin may exert protective effects through immunomodulatory mechanisms [41]. Furthermore, adipose tissue participates in renin-angiotensin system activation, potentially improving hemodynamic stability, whereas obesity may reduce muscle catabolism and help preserve muscle function [42]. In critically ill people, body weight fluctuations are influenced by multiple factors including fluid balance, metabolic stress, and nutritional interventions. Although fluid overload is associated with short-term weight gain and increased mortality risk [43], studies have demonstrated only weak correlations between changes in BMI and net fluid intake, suggesting that BMI may more reliably reflect overall patient status and that its prognostic value in critical illness remains relatively independent of fluid balance considerations [44].

Our subgroup analyses demonstrated that the TyG-BMI exhibited more substantial protective effects in critically ill ASCVD patients without concurrent heart failure, atrial fibrillation, diabetes mellitus, or chronic kidney disease. This phenomenon may be attributable to the relatively better glycemic control observed in patients without these comorbidities, as supported by the literature, which confirms a strong interrelationship between heart failure, atrial fibrillation, renal dysfunction and diabetes mellitus, with these patients demonstrating a higher likelihood of comorbid diabetes that significantly impacts metabolic profiles [45–47]. A pivotal study by Wang et al. demonstrated that compared with obesity, type 2 diabetes mellitus has stronger predictive value for mortality, potentially neutralizing the survival advantages linked to excess adiposity [48]; this finding may explain the weakened TyG-BMI-mortality relationship observed in patients with these comorbid conditions. Taken together, the results of this investigation offer a thorough analysis of the association of the TyG-BMI with both acute and chronic outcomes in severely ill ASCVD patients, revealing a unique L-curve relationship. These findings not only highlight the differential prognostic implications of various insulin resistance indices across distinct patient populations but also emphasize the clinical necessity of implementing personalized therapeutic strategies tailored to individual metabolic characteristics and comorbidity patterns.

## Strengths and limitations

This investigation has several notable strengths that merit emphasis. Foremost, this study constitutes an inaugural systematic assessment of the ability of the TyG-BMI to predict mortality in ASCVD patients, with a special emphasis on critically ill elderly individuals, offering new insights to guide therapeutic approaches for this high-risk population. Moreover, employing an extensive dataset with complete outcome tracking markedly improves the generalizability and dependability of our conclusions. Certain limitations must be recognized. First, the MIMIC-IV database originates from a single institution, which may limit the generalizability of our findings to broader populations and precludes in-depth analysis of specific indications for hospitalization and pathways to mortality. Second, the retrospective study design inherently has potential biases, including inconsistencies in data coding, incomplete documentation, and possible misclassification bias. Third, the TyG-BMI in this study was calculated using glucose and triglyceride levels measured from routine blood draws within 24 h of ICU admission rather than under strict fasting conditions. This may lead to a systematic overestimation of the TyG-BMI, thereby introducing potential bias. Fourth, although we attempted to control for bias through subgroup analyses and medication adjustment, the potential misclassification of the TyG-BMI due to glucose-lowering therapy cannot be entirely ruled out. Fifth, this study excluded patients who died within 24 h of admission or who had an ICU stay of less than 24 h. This may have led to an underestimation of the true mortality rate, affected the accuracy of the estimated exposure–outcome association, and limited the generalizability of our findings to the highest-risk population of patients who died during the hyperacute phase. Finally, the observed strong inverse association may be partly attributable to unmeasured confounders including frailty, sarcopenia, and cachexia, which are closely linked to metabolic reserve and muscle wasting. Future research should include direct assessments of these factors to better delineate their role in the relationship between TyG-BMI and prognosis. These methodological considerations collectively underscore the necessity for future multicenter prospective studies to validate our conclusions and strengthen the evidence base through more rigorous study designs.

## Conclusion

Among critically ill ASCVD patients, the TyG-BMI is significantly associated with both short- and long-term mortality outcomes according to the characteristic L-curve. Although causality cannot be established because of the observational study design, this unique nonlinear pattern suggests that incorporating the TyG-BMI into existing prognostic frameworks may provide useful metabolic information to support risk stratification and inform therapeutic decision-making in critical care settings.

## Supplementary Information

Supplementary Material 1.

## Data Availability

The datasets generated and analyzed in this investigation are available from the corresponding author pending appropriate scientific justification and approval.
