# Supplementary material for: Association between triglyceride glucose-body mass index and all-cause mortality in critically ill patients with atherosclerotic cardiovascular diseases: a retrospective cohort study using the MIMIC-IV database
Source: BMC Cardiovasc Disord. 2026 May 29;26:646. doi: 10.1186/s12872-026-06022-1 (PMC13418136; doi:10.1186/s12872-026-06022-1)
Supplement: Supplementary file 1 — Supplementary Material 1. [file 12872_2026_6022_MOESM1_ESM.docx]

**Association between ​triglyceride glucose-body mass index and all-cause mortality in critically ill patients with atherosclerotic cardiovascular diseases: a retrospective cohort study using the MIMIC-IV database**

**Supplementary materials**

**Table S1** The meaning of ICD-9 and ICD-10 codes for diseases

**Table S2** The variance inflation factor (VIF) of variables

**Table S3** Comparison of baseline characteristics between included and excluded populations

**Table S4** HRs (95% CIs) for 90-d and 180-d mortality according to TyG-BMI quartiles

**Table S5** TyG-BMI quartile-based HRs (95% CIs) for mortality following the removal of individuals with missing baseline variables

**Table S6** TyG-BMI quartile-based HRs (95% CIs) for mortality following the removal of individuals with self-reported cancer at baseline

**Table S7** TyG-BMI quartile-based HRs (95% CIs) for mortality after removing extreme TyG-BMI values

**Table S8** Threshold analyses of TyG-BMI on outcome using two-piecewise regression models

**Table S9** Incremental value of TyG-BMI for 90-d and 180-d mortality

**Figure S1** Flowchart of subject screening. Flow chart illustrating the inclusion and exclusion criteria

**Figure S2** Kaplan-Meier survival curve for 90-d and 180-d mortality according to TyG-BMI

**Figure S3** The potential non-linear relationship between TyG-BMI and 90-/180-d mortality in patients with ASCVD was evaluated using a restricted cubic spline analysis

**Figure S4** ROC curve analysis of the incremental effect of TyG-BMI on 90-d all-cause mortality

**Figure S5** ROC curve analysis of the incremental effect of TyG-BMI on 180-d all-cause mortality

**Figure S6** Subgroup analyses for the correlation of TyG-BMI with risk of 90-d and 180-d mortality in ASCVD patients

**Table S1 The meaning of ICD-9 and ICD-10 codes for diseases**

| Ischemic | ICD9 | 433.00,433.01,433.10,433.11,433.20,433.21,433.30,433.31,4 |
| --- | --- | --- |
| stroke |  | 33.80,433.81,433.90,433.91,434.00,434.01,434.10,434.11,43 |
|  |  | 4.90,434.91 |
|  | ICD10 | I63.41, I63.42, I63.43, I63.44, I63.5, I63.51, I63.519, I63.52, |
|  |  | I63.53, I63.54, |
|  |  | I65, I66, I63.40, I63.411, I63.412, I63.413, I63.419, I63.421, |
|  |  | I63.422, I63.423, |
|  |  | I63.429, I63.431, I63.432, I63.433, I63.439, I63.441, |
|  |  | I63.442, I63.443, I63.449, |
|  |  | I63.9, I63.50, I63.511, I63.512, I63.513, I63.521, I63.522, |
|  |  | I63.523, I63.529, |
|  |  | I63.531, I63.532, I63.533, I63.539, I63.541, I63.542, |
|  |  | I63.543, I63.549, I63.9, |
|  |  | I63.6, I63.8, I63.81, I63.89, I63.9 |
| Ischemic | ICD10 | I25.5, I20.0, I20.1, I20.8, I20.9, I21.0, I21.01, I21.02, I21.09, |
| heart |  | I21.11, I21.19, |
| disease |  | I21.21, I21.29, I21.3, I21.4, I21.9, I21.A1, I21.A9, I22.0, |
|  |  | I22.1, I22.2, I22.8, |
|  |  | I22.9, I23.0, I23.1, I23.2, I23.3, I23.4, I23.5, I23.6, I23.7, |
|  |  | I23.8, I24.0, I24.1, |
|  |  | I24.8, I24.9, I25.10, I25.110, I25.111, I25.118, I25.119, |
|  |  | I25.810, I25.82, I25.83, |
|  |  | I25.84 |

ICD: international classification of diseases

**Table S2 The variance inflation factor (VIF) of variables**

| Variables | VIF |
| --- | --- |
| Age,years | 1.064 |
| Gender, n (%) | 1.067 |
| Hemoglobin(g/dL) | 1.473 |
| White blood cell(K/µL) | 1.095 |
| Albumin(g/dL) | 1.593 |
| BUN(mg/dL) | 2.284 |
| Creatinine(mg/dL) | 2.367 |
| Sodium(mEq/L) | 2.223 |
| Potassium(mEq/L) | 1.284 |
| Chloride(mEq/L) | 2.311 |
| SBP,mmHg | 1.541 |
| DBP,mmHg | 1.505 |
| Heart Rate,bpm | 1.299 |
| RR, times/min | 1.187 |
| Congestive Heart Failure, n (%) | 1.653 |
| COPD, n (%) | 1.066 |
| Atrial Fibrillation, n (%) | 1.083 |
| Malignant Cancer, n (%) | 1.065 |
| Diabetes, n (%) | 1.293 |
| Hypertension, n (%) | 1.388 |
| Antiplatelet, n (%) | 1.313 |
| Statin, n (%) | 1.408 |
| Insulin, n (%) | 1.236 |
| Diuretics, n (%) | 1.239 |

**Table S3 Comparison of baseline characteristics between included and excluded populations**

| **Variable** | **Included (N=1637)** | **Excluded(N=12759)** | ***P* value** |
| --- | --- | --- | --- |
| Age,years | 68.3(59.7,77.7) | 67.8 (58.9, 76.5) | 0.283 |
| Gender, n (%) |  |  | 0.344 |
| Female | 561(34.3) | 550 (33.6) |  |
| Male | 1076(65.7) | 1080 (66.4) |  |
| Ethnicity, n (%) |  |  | 0.581 |
| White | 915(55.9) | 905 (55.3) |  |
| Black | 122(7.4) | 120 (7.3) |  |
| Other | 600(36.7) | 590 (37.4) |  |
| Hospital LOS,days | 9.0(5.1,15.0) | 9.2 (5.3, 14.8) | 0.617 |
| ICU LOS,days | 3.1(1.5,6.8) | 3.3 (1.6, 6.5) | 0.134 |
| Laboratory parameters |  |  |  |
| Hemoglobin(g/dL) | 11.7(10.0,13.2) | 11.5 (9.8, 13.0) | 0.872 |
| White blood cell(K/µL) | 11.6(8.9,15.1) | 11.8 (9.1, 14.9) | 0.409 |
| Lymphocytes(K/µL) | 1.4(0.9,2.1) | 1.5 (1.0, 2.0) | 0.725 |
| Neutrophils(K/µL) | 9.0(6.2,12.7) | 8.8 (6.0, 12.5) | 0.326 |
| Platelets(K/µL) | 194.0(149.0,247.0) | 192.0 (147.0, 245.0) | 0.568 |
| BUN(mg/dL) | 18.0(13.5,27.5) | 17.5 (13.0, 26.0) | 0.195 |
| Creatinine(mg/dL) | 1.0(0.8,1.4) | 1.1 (0.9, 1.3) | 0.742 |
| ALT(IU/L) | 28.0(17.0,62.5) | 29.0 (18.0, 60.0) | 0.503 |
| AST(IU/L) | 42.0(24.0,117.5) | 43.0 (25.0, 115.0) | 0.089 |
| Albumin(g/dL) | 3.5(3.0,3.9) | 3.4 (3.1, 3.8) | 0.654 |
| TC(mg/dL) | 158.0(126.0,194.0) | 156.0 (124.0, 192.0) | 0.377 |
| HDL(mg/dL) | 44.0(35.0,55.0) | 43.0 (34.0, 54.0) | 0.821 |
| LDL(mg/dL) | 84.0(60.0,115.0) | 82.0 (58.0, 113.0) | 0.246 |
| Bicarbonate(mEq/L) | 22.5(20.5,24.0) | 22.0 (20.0, 23.5) | 0.598 |
| Sodium(mEq/L) | 138.5(136.0,141.0) | 138.0 (135.5, 140.5) | 0.932 |
| Potassium(mEq/L) | 4.3(4.0,4.6) | 4.2 (3.9, 4.5) | 0.471 |
| Chloride(mEq/L) | 104.0(101.0,107.0) | 103.0 (100.0, 106.0) | 0.715 |
| Aniongap(mEq/L) | 13.5(11.0,16.0) | 13.0 (10.5, 15.5) | 0.153 |
| INR | 1.3(1.1,1.4) | 1.2 (1.0, 1.3) | 0.689 |
| PT(s) | 13.5(12.2,15.4) | 13.0 (12.0, 15.0) | 0.362 |
| PTT(s) | 32.7(27.8,49.3) | 33.0 (28.0, 48.0) | 0.794 |
| TyG-BMI | 337.4(297.4,433.0) | 335.0 (295.0, 430.0) | 0.527 |
| Vital signs |  |  |  |
| SBP,mmHg | 116.3(107.2,130.2) | 115.0 (106.0, 129.0) | 0.218 |
| DBP,mmHg | 63.8(57.2,73.1) | 64.0 (56.5, 72.5) | 0.846 |
| MBP,mmHg | 79.4(73.7,88.5) | 78.0 (72.0, 87.0) | 0.431 |
| Heart Rate,bpm | 80.3(71.9,89.9) | 79.0 (71.0, 88.5) | 0.673 |
| RR, times/min | 18.9(17.0,21.5) | 18.5 (16.5, 21.0) | 0.312 |
| Disease severity scoring system |  |  |  |
| SOFA | 4.0(2.0,7.0) | 4.5 (2.5, 6.5) | 0.759 |
| SIRS | 3.0(2.0,3.0) | 3.0 (2.0, 3.0) | 0.185 |
| SAPS II | 35.0(27.0,44.0) | 34.0 (26.0, 43.0) | 0.638 |
| APS III | 37.0(28.0,53.0) | 36.0 (27.0, 52.0) | 0.497 |
| OASIS | 33.0(27.0,39.0) | 32.0 (26.0, 38.0) | 0.907 |
| Medications, n (%) |  |  |  |
| Antiplatelet | 1366(83.4) | 1350 (82.5) | 0.127 |
| Statin | 1351(82.5) | 1340 (81.9) | 0.778 |
| Insulin | 1264(77.2) | 1250 (76.4) | 0.423 |
| Beta blockers | 1182(72.2) | 1170 (71.5) | 0.699 |
| ACEI/ARB | 1414(86.4) | 1400 (85.5) | 0.267 |
| Diuretics | 1089(66.5) | 1075 (65.7) | 0.813 |
| Heparin | 1548(94.6) | 1530 (93.5) | 0.536 |
| Warfarin | 265(16.2) | 260 (15.9) | 0.172 |
| Comorbidities |  |  |  |
| Congestive Heart Failure, n (%) | 618(37.8) | 610 (37.3) | 0.627 |
| COPD, n (%) | 300(18.3) | 295 (18.0) | 0.398 |
| Renal Disease, n (%) | 326(19.9) | 320 (19.6) | 0.885 |
| Malignant Cancer, n (%) | 94(5.7) | 90 (5.5) | 0.301 |
| Liver Disease, n (%) | 88(5.4) | 85 (5.2) | 0.553 |
| Diabetes, n (%) | 586(35.8) | 580 (35.4) | 0.206 |
| Hypertension, n (%) | 755(46.1) | 745 (45.5) | 0.731 |
| Atrial Fibrillation, n (%) | 601(36.7) | 595 (36.4) | 0.487 |
| Outcome, n (%) |  |  |  |
| 30-d mortality | 315(19.2) | 310 (18.9) | 0.943 |
| 90-d mortality | 366(22.4) | 360 (22.0) | 0.357 |
| 180-d mortality | 408(24.9) | 400 (24.4) | 0.662 |
| 1 year mortality | 453(27.7) | 445 (27.2) | 0.239 |
| RRT, n (%) | 171(10.4) | 170 (10.4) | 0.512 |
| Ventilation, n (%) | 1375(84.0) | 1360 (83.1) | 0.876 |

LOS: Length of Stay; BUN: blood urea nitrogen; ALT: alanine transaminase; AST: aspartate aminotransferase; TC: total cholesterol; HDL: high-density lipoprotein; LDL: low-density lipoprotein; INR: international normalized ratio; PT: prothrombin time; PTT: partial thromboplastin time; TyG-BMI: ​triglyceride glucose-body mass index​​; SBP: systolic blood pressure; DBP: diastolic blood pressure; MBP: mean blood pressure; RR: respiratory rate; SOFA: Sequential Organ Failure Assessment; SIRS: Systemic Inflammatory Response Syndrome; SAPS II: Simplified Acute Physiology Score II; APS III: Acute Physiology Score III; OASIS: Oxford Acute Severity of Illness Score; COPD: chronic obstructive pulmonary disease

**Table S4 HRs (95% CIs) for 90-d and 180-d mortality according to TyG-BMI quartiles**

| **Variable** | **Model I** | | **Model II** | | **Model III** | | **Model IV** | |
| --- | --- | --- | --- | --- | --- | --- | --- | --- |
|  | **HR (95% CI)** | ***P* value** | **HR (95% CI)** | ***P* value** | **HR (95% CI)** | ***P* value** | **HR (95% CI)** | ***P* value** |
| **90-d mortality** |  |  |  |  |  |  |  |  |
| TyG-BMI quantile |  |  |  |  |  |  |  |  |
| Q1 | Ref. |  | Ref. |  | Ref. |  | Ref. |  |
| Q2 | 0.46(0.36,0.59) | <0.001 | 0.47(0.37,0.60) | <0.001 | 0.64(0.47,0.89) | 0.007 | 0.61(0.44,0.84) | 0.003 |
| Q3 | 0.27(0.21,0.37) | <0.001 | 0.27(0.20,0.37) | <0.001 | 0.43(0.30,0.61) | <0.001 | 0.41(0.28,0.58) | <0.001 |
| Q4 | 0.12(0.08,0.18) | <0.001 | 0.12(0.08,0.18) | <0.001 | 0.15(0.10,0.24) | <0.001 | 0.14(0.09,0.22) | <0.001 |
| P for trend |  | <0.001 |  | <0.001 |  | <0.001 |  | <0.001 |
| **180-d mortality** |  |  |  |  |  |  |  |  |
| TyG-BMI quantile |  |  |  |  |  |  |  |  |
| Q1 | Ref. |  | Ref. |  | Ref. |  | Ref. |  |
| Q2 | 0.48(0.38,0.60) | <0.001 | 0.49(0.38,0.61) | <0.001 | 0.65(0.48,0.88) | 0.005 | 0.62(0.45,0.84) | 0.002 |
| Q3 | 0.29(0.22,0.38) | <0.001 | 0.29(0.22,0.38) | <0.001 | 0.40(0.29,0.57) | <0.001 | 0.38(0.27,0.54) | <0.001 |
| Q4 | 0.13(0.09,0.19) | <0.001 | 0.13(0.09,0.19) | <0.001 | 0.16(0.10,0.24) | <0.001 | 0.14(0.09,0.21) | <0.001 |
| *P* for trend |  | <0.001 |  | <0.001 |  | <0.001 |  | <0.001 |

Model I: no adjustment

Model II: adjusted for age, sex and race

Model III: further adjusted for SBP, DBP, heart rate, respiratory rate, hemoglobin, white blood cell, albumin, BUN, creatinine, sodium, potassium, chloride

Model IV: further adjusted for congestive heart failure, COPD, malignant cancer, diabetes, hypertension, atrial fibrillation, antiplatelet, statin, insulin, diuretics

**Table S5 TyG-BMI quartile-based HRs (95% CIs) for mortality following the removal of individuals with missing baseline variables**

| **Variable** | **Model I** | | **Model II** | | **Model III** | | **Model IV** | |
| --- | --- | --- | --- | --- | --- | --- | --- | --- |
|  | **HR (95% CI)** | **P value** | **HR (95% CI)** | **P value** | **HR (95% CI)** | **P value** | **HR (95% CI)** | **P value** |
| **30-d mortality** |  |  |  |  |  |  |  |  |
| TyG-BMI quantile |  |  |  |  |  |  |  |  |
| Q1 | Ref. |  | Ref. |  | Ref. |  | Ref. |  |
| Q2 | 0.41(0.32,0.54) | <0.001 | 0.42(0.32,0.55) | <0.001 | 0.61(0.44,0.87) | 0.005 | 0.59(0.42,0.83) | 0.003 |
| Q3 | 0.26(0.19,0.36) | <0.001 | 0.26(0.19,0.35) | <0.001 | 0.42(0.29,0.61) | <0.001 | 0.41(0.28,0.60) | <0.001 |
| Q4 | 0.11(0.07,0.17) | <0.001 | 0.11(0.07,0.17) | <0.001 | 0.13(0.07,0.21) | <0.001 | 0.12(0.07,0.22) | <0.001 |
| P for trend |  | <0.001 |  | <0.001 |  | <0.001 |  | <0.001 |
| **90-d mortality** |  |  |  |  |  |  |  |  |
| TyG-BMI quantile |  |  |  |  |  |  |  |  |
| Q1 | Ref. |  | Ref. |  | Ref. |  | Ref. |  |
| Q2 | 0.45(0.35,0.58) | <0.001 | 0.46(0.36,0.59) | <0.001 | 0.63(0.46,0.87) | 0.005 | 0.61(0.44,0.84) | 0.003 |
| Q3 | 0.27(0.20,0.37) | <0.001 | 0.27(0.20,0.36) | <0.001 | 0.43(0.31,0.61) | <0.001 | 0.42(0.29,0.60) | <0.001 |
| Q4 | 0.12(0.08,0.18) | <0.001 | 0.12(0.08,0.19) | <0.001 | 0.16(0.10,0.24) | <0.001 | 0.14(0.09,0.23) | <0.001 |
| **180-d mortality** |  |  |  |  |  |  |  |  |
| TyG-BMI quantile |  |  |  |  |  |  |  |  |
| Q1 | Ref. |  | Ref. |  | Ref. |  | Ref. |  |
| Q2 | 0.47(0.37,0.59) | <0.001 | 0.47(0.38,0.60) | <0.001 | 0.64(0.47,0.87) | 0.007 | 0.61(0.45,0.83) | 0.002 |
| Q3 | 0.29(0.22,0.38) | <0.001 | 0.28(0.22,0.38) | <0.001 | 0.41(0.29,0.57) | <0.001 | 0.39(0.28,0.56) | <0.001 |
| Q4 | 0.13(0.09,0.19) | <0.001 | 0.13(0.09,0.19) | <0.001 | 0.16(0.10,0.24) | <0.001 | 0.14(0.09,0.22) | <0.001 |
| P for trend |  | <0.001 |  | <0.001 |  | <0.001 |  | <0.001 |
| **1 year mortality** |  |  |  |  |  |  |  |  |
| TyG-BMI quantile |  |  |  |  |  |  |  |  |
| Q1 | Ref. |  | Ref. |  | Ref. |  | Ref. |  |
| Q2 | 0.50(0.40,0.62) | <0.001 | 0.50(0.40,0.63) | <0.001 | 0.65(0.49,0.88) | 0.007 | 0.63(0.47,0.84) | 0.002 |
| Q3 | 0.30(0.23,0.39) | <0.001 | 0.29(0.23,0.38) | <0.001 | 0.41(0.30,0.57) | <0.001 | 0.40(0.29,0.56) | <0.001 |
| Q4 | 0.13(0.09,0.19) | <0.001 | 0.13(0.09,0.19) | <0.001 | 0.15(0.10,0.23) | <0.001 | 0.13(0.09,0.20) | <0.001 |
| *P* for trend |  | <0.001 |  | <0.001 |  | <0.001 |  | <0.001 |

Model I: no adjustment

Model II: adjusted for age, sex and race

Model III: further adjusted for SBP, DBP, heart rate, respiratory rate, hemoglobin, white blood cell, albumin, BUN, creatinine, sodium, potassium, chloride

Model IV: further adjusted for congestive heart failure, COPD, malignant cancer, diabetes, hypertension, atrial fibrillation, antiplatelet, statin, insulin, diuretics

**Table S6 TyG-BMI quartile-based HRs (95% CIs) for mortality following the removal of individuals with self-reported cancer at baseline**

| **Variable** | **Model I** | | **Model II** | | **Model III** | | **Model IV** | |
| --- | --- | --- | --- | --- | --- | --- | --- | --- |
|  | **HR (95% CI)** | **P value** | **HR (95% CI)** | **P value** | **HR (95% CI)** | **P value** | **HR (95% CI)** | **P value** |
| **30-d mortality** |  |  |  |  |  |  |  |  |
| TyG-BMI quantile |  |  |  |  |  |  |  |  |
| Q1 | Ref. |  | Ref. |  | Ref. |  | Ref. |  |
| Q2 | 0.45(0.34,0.60) | <0.001 | 0.45(0.34,0.60) | <0.001 | 0.65(0.45,0.92) | 0.016 | 0.62(0.43,0.89) | 0.010 |
| Q3 | 0.27(0.19,0.37) | <0.001 | 0.27(0.19,0.37) | <0.001 | 0.43(0.30,0.64) | <0.001 | 0.41(0.28,0.62) | <0.001 |
| Q4 | 0.11(0.07,0.17) | <0.001 | 0.11(0.07,0.17) | <0.001 | 0.11(0.06,0.19) | <0.001 | 0.10(0.05,0.17) | <0.001 |
| P for trend |  | <0.001 |  | <0.001 |  | <0.001 |  | <0.001 |
| **90-d mortality** |  |  |  |  |  |  |  |  |
| TyG-BMI quantile |  |  |  |  |  |  |  |  |
| Q1 | Ref. |  | Ref. |  | Ref. |  | Ref. |  |
| Q2 | 0.49(0.38,0.64) | <0.001 | 0.50(0.38,0.65) | <0.001 | 0.69(0.49,0.96) | 0.027 | 0.66(0.47,0.93) | 0.018 |
| Q3 | 0.29(0.21,0.39) | <0.001 | 0.29(0.21,0.39) | <0.001 | 0.45(0.31,0.65) | <0.001 | 0.43(0.30,0.64) | <0.001 |
| Q4 | 0.12(0.08,0.19) | <0.001 | 0.12(0.08,0.19) | <0.001 | 0.13(0.08,0.22) | <0.001 | 0.11(0.07,0.19) | <0.001 |
| **180-d mortality** |  |  |  |  |  |  |  |  |
| TyG-BMI quantile |  |  |  |  |  |  |  |  |
| Q1 | Ref. |  | Ref. |  | Ref. |  | Ref. |  |
| Q2 | 0.51(0.40,0.66) | <0.001 | 0.51(0.40,0.66) | <0.001 | 0.69(0.50,0.95) | 0.023 | 0.66(0.48,0.91) | 0.012 |
| Q3 | 0.31(0.23,0.41) | <0.001 | 0.30(0.23,0.40) | <0.001 | 0.43(0.30,0.61) | <0.001 | 0.40(0.28,0.58) | <0.001 |
| Q4 | 0.13(0.09,0.19) | <0.001 | 0.13(0.09,0.19) | <0.001 | 0.14(0.09,0.22) | <0.001 | 0.12(0.07,0.19) | <0.001 |
| P for trend |  | <0.001 |  | <0.001 |  | <0.001 |  | <0.001 |
| **1 year mortality** |  |  |  |  |  |  |  |  |
| TyG-BMI quantile |  |  |  |  |  |  |  |  |
| Q1 | Ref. |  | Ref. |  | Ref. |  | Ref. |  |
| Q2 | 0.54(0.43,0.68) | <0.001 | 0.54(0.43,0.68) | <0.001 | 0.71(0.52,0.96) | 0.024 | 0.68(0.50,0.92) | 0.013 |
| Q3 | 0.31(0.24,0.41) | <0.001 | 0.31(0.23,0.41) | <0.001 | 0.43(0.31,0.61) | <0.001 | 0.41(0.29,0.58) | <0.001 |
| Q4 | 0.13(0.09,0.19) | <0.001 | 0.13(0.09,0.19) | <0.001 | 0.14(0.09,0.22) | <0.001 | 0.11(0.07,0.17) | <0.001 |
| *P* for trend |  | <0.001 |  | <0.001 |  | <0.001 |  | <0.001 |

Model I: no adjustment

Model II: adjusted for age, sex and race

Model III: further adjusted for SBP, DBP, heart rate, respiratory rate, hemoglobin, white blood cell, albumin, BUN, creatinine, sodium, potassium, chloride

Model IV: further adjusted for congestive heart failure, COPD, malignant cancer, diabetes, hypertension, atrial fibrillation, antiplatelet, statin, insulin, diuretics

**Table S7 TyG-BMI quartile-based HRs (95% CIs) for mortality after removing extreme TyG-BMI values**

| **Variable** | **Model I** | | **Model II** | | **Model III** | | **Model IV** | |
| --- | --- | --- | --- | --- | --- | --- | --- | --- |
|  | **HR (95% CI)** | **P value** | **HR (95% CI)** | **P value** | **HR (95% CI)** | **P value** | **HR (95% CI)** | **P value** |
| **30-d mortality** |  |  |  |  |  |  |  |  |
| TyG-BMI quantile |  |  |  |  |  |  |  |  |
| Q1 | Ref. |  | Ref. |  | Ref. |  | Ref. |  |
| Q2 | 0.43(0.33,0.57) | <0.001 | 0.44(0.33,0.57) | <0.001 | 0.64(0.45,0.90) | 0.010 | 0.60(0.42,0.85) | 0.004 |
| Q3 | 0.27(0.19,0.36) | <0.001 | 0.26(0.19,0.36) | <0.001 | 0.43(0.29,0.62) | <0.001 | 0.40(0.27,0.59) | <0.001 |
| Q4 | 0.11(0.07,0.17) | <0.001 | 0.11(0.07,0.17) | <0.001 | 0.13(0.07,0.22) | <0.001 | 0.11(0.06,0.19) | <0.001 |
| P for trend |  | <0.001 |  | <0.001 |  | <0.001 |  | <0.001 |
| **90-d mortality** |  |  |  |  |  |  |  |  |
| TyG-BMI quantile |  |  |  |  |  |  |  |  |
| Q1 | Ref. |  | Ref. |  | Ref. |  | Ref. |  |
| Q2 | 0.47(0.37,0.60) | <0.001 | 0.48(0.37,0.61) | <0.001 | 0.65(0.47,0.90) | 0.010 | 0.63(0.45,0.87) | 0.005 |
| Q3 | 0.28(0.21,0.37) | <0.001 | 0.28(0.21,0.37) | <0.001 | 0.44(0.31,0.62) | <0.001 | 0.42(0.29,0.60) | <0.001 |
| Q4 | 0.12(0.08,0.18) | <0.001 | 0.12(0.08,0.18) | <0.001 | 0.15(0.09,0.24) | <0.001 | 0.14(0.08,0.22) | <0.001 |
| **180-d mortality** |  |  |  |  |  |  |  |  |
| TyG-BMI quantile |  |  |  |  |  |  |  |  |
| Q1 | Ref. |  | Ref. |  | Ref. |  | Ref. |  |
| Q2 | 0.49(0.38,0.61) | <0.001 | 0.49(0.39,0.62) | <0.001 | 0.66(0.49,0.90) | 0.008 | 0.63(0.46,0.86) | 0.003 |
| Q3 | 0.29(0.22,0.39) | <0.001 | 0.29(0.22,0.38) | <0.001 | 0.41(0.29,0.58) | <0.001 | 0.39(0.28,0.56) | <0.001 |
| Q4 | 0.13(0.09,0.19) | <0.001 | 0.13(0.09,0.19) | <0.001 | 0.15(0.10,0.24) | <0.001 | 0.14(0.09,0.22) | <0.001 |
| P for trend |  | <0.001 |  | <0.001 |  | <0.001 |  | <0.001 |
| **1 year mortality** |  |  |  |  |  |  |  |  |
| TyG-BMI quantile |  |  |  |  |  |  |  |  |
| Q1 | Ref. |  | Ref. |  | Ref. |  | Ref. |  |
| Q2 | 0.51(0.41,0.64) | <0.001 | 0.52(0.41,0.64) | <0.001 | 0.67(0.50,0.90) | 0.007 | 0.65(0.48,0.87) | 0.003 |
| Q3 | 0.30(0.23,0.39) | <0.001 | 0.30(0.23,0.38) | <0.001 | 0.41(0.30,0.57) | <0.001 | 0.40(0.29,0.56) | <0.001 |
| Q4 | 0.13(0.09,0.18) | <0.001 | 0.12(0.09,0.18) | <0.001 | 0.14(0.09,0.21) | <0.001 | 0.12(0.08,0.19) | <0.001 |
| *P* for trend |  | <0.001 |  | <0.001 |  | <0.001 |  | <0.001 |

Model I: no adjustment

Model II: adjusted for age, sex and race

Model III: further adjusted for SBP, DBP, heart rate, respiratory rate, hemoglobin, white blood cell, albumin, BUN, creatinine, sodium, potassium, chloride

Model IV: further adjusted for congestive heart failure, COPD, malignant cancer, diabetes, hypertension, atrial fibrillation, antiplatelet, statin, insulin, diuretics

**Table S8 Threshold analyses of TyG-BMI on outcome using two-piecewise regression models**

|  | **Adjusted HR (95%CI)** | ***P* value** |
| --- | --- | --- |
| **90-d mortality** |  |  |
| Fitting by the standard linear model | 0.990(0.989,0.992) | <0.001 |
| Fitting by the two-piecewise linear model |  |  |
| Inflection point | 446.3 |  |
| TyG-BMI<446.3 | 0.988(0.987-0.990) | <0.001 |
| TyG-BMI>446.3 | 1.003(1.000-1.007) | 0.036 |
| *P* for Log-likelihood ratio |  | <0.001 |
| **180-d mortality** |  |  |
| Fitting by the standard linear model | 0.991(0.989-0.992) | <0.001 |
| Fitting by the two-piecewise linear model |  |  |
| Inflection point | 444.4 |  |
| TyG-BMI<444.4 | 0.989(0.987-0.990) | <0.001 |
| TyG-BMI>444.4 | 1.003(1.000-1.006) | 0.071 |
| *P* for Log-likelihood ratio |  | <0.001 |

TyG-BMI: ​triglyceride glucose-body mass index​​

**Table S9 Incremental value of TyG-BMI for 90-d and 180-d mortality**

|  | **C-statistic^a^** | **C-statistic^b^** | **Δ C-statistic** | **P-value for Δ C** | **IDI (95% CI)** | **NRI (95% CI)** |
| --- | --- | --- | --- | --- | --- | --- |
| **90 day-mortality** |  |  |  |  |  |  |
| SOFA + TyG-BMI vs. SOFA | 0.64(0.59,0.69) | 0.75(0.72,0.79) | 0.11 | <0.001 | 0.15(0.03,0.17) | 0.32(0.06,0.39) |
| SAPS II + TyG-BMI vs. SAPS II | 0.73(0.69,0.77) | 0.80(0.76,0.83) | 0.07 | <0.001 | 0.11(0.02,0.13) | 0.27(0.05,0.37) |
| APS III + TyG-BMI vs. APS III | 0.69(0.65,0.74) | 0.79(0.75,0.82) | 0.10 | <0.001 | 0.13(0.02,0.17) | 0.30(0.05,0.43) |
| OASIS + TyG-BMI vs. OASIS | 0.70(0.66,0.74) | 0.78(0.74,0.82) | 0.08 | <0.001 | 0.14(0.03,0.19) | 0.31(0.05,0.37) |
| **180 day-mortality** |  |  |  |  |  |  |
| SOFA + TyG-BMI vs. SOFA | 0.63(0.59,0.68) | 0.75(0.71,0.78) | 0.12 | <0.001 | 0.16(0.03,0.21) | 0.32(0.06,0.42) |
| SAPS II + TyG-BMI vs. SAPS II | 0.72(0.68,0.76) | 0.79(0.75,0.82) | 0.07 | <0.001 | 0.12(0.02,0.17) | 0.29(0.06,0.39) |
| APS III + TyG-BMI vs. APS III | 0.68(0.64,0.73) | 0.78(0.74,0.81) | 0.10 | <0.001 | 0.15(0.03,0.19) | 0.31(0.07,0.39) |
| OASIS + TyG-BMI vs. OASIS | 0.69(0.65,0.73) | 0.77(0.73,0.80) | 0.08 | <0.001 | 0.14(0.02,0.18) | 0.31(0.06,0.39) |

SOFA: Sequential Organ Failure Assessment; SAPS II: Simplified Acute Physiology Score II; APS III: Acute Physiology Score III; OASIS: Oxford Acute Severity of Illness Score; FI-LAB: Frailty Index based on Laboratory; TyG-BMI: ​triglyceride glucose-body mass index​​

**Figure S1 Flow chart illustrating the inclusion and exclusion criteria**


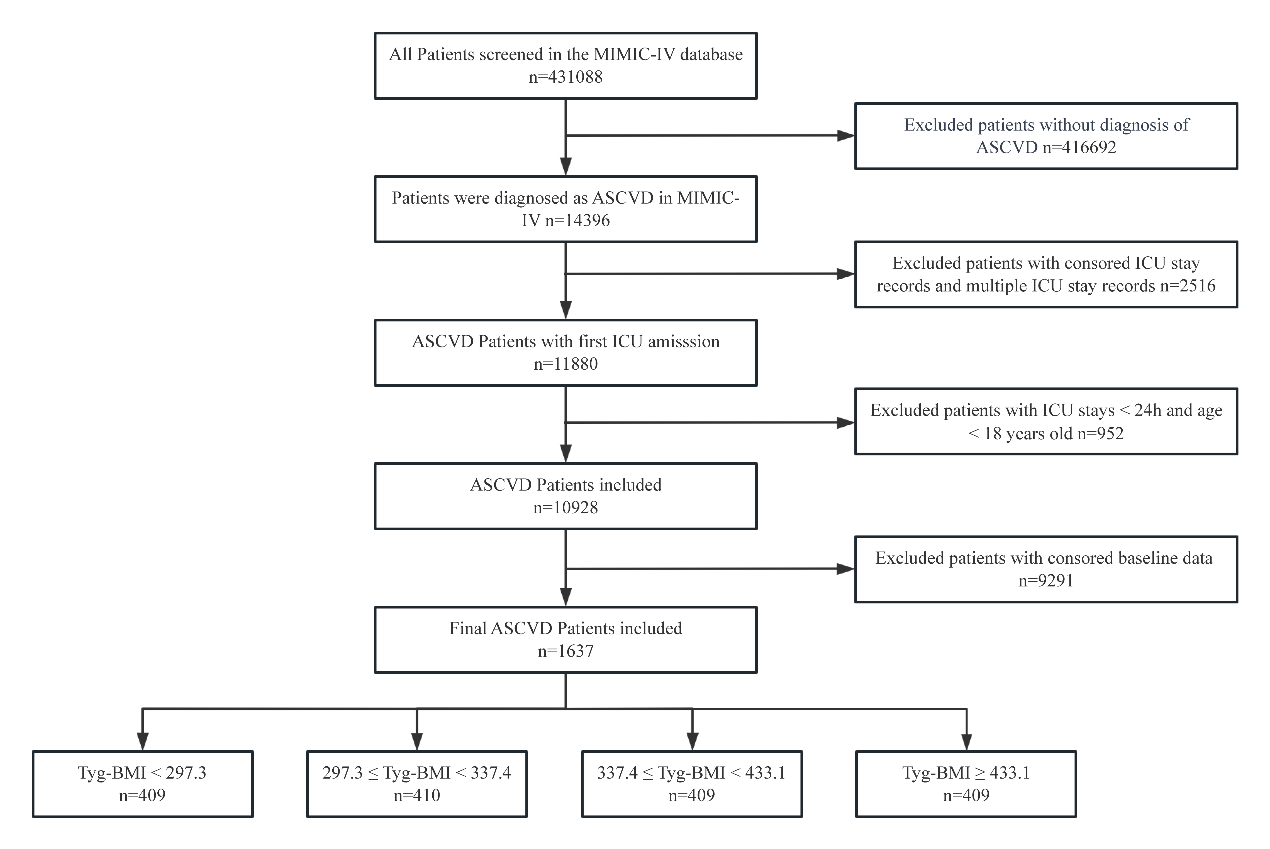


ASCVD: atherosclerotic cardiovascular diseases; ICU: intensive care unit; MIMIC: Medical Information Mart for Intensive Care.

**Figure S2 Kaplan-Meier survival curve for 90-d and 180-d mortality according to TyG-BMI**

**
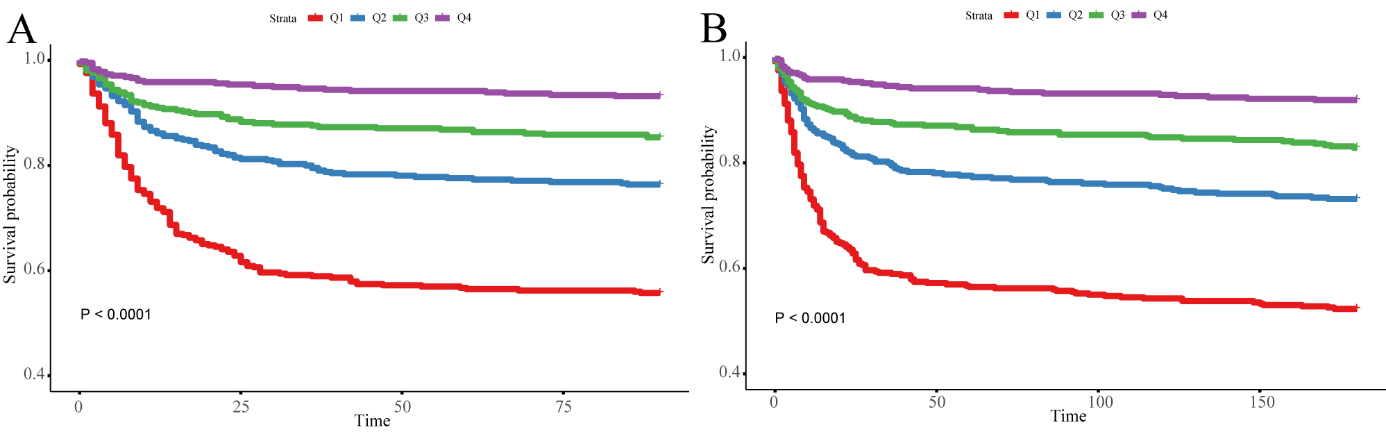
**

(A) Kaplan-Meier survival curve for 90-d mortality according to TyG-BMI; (B) Kaplan-Meier survival curve for 180-d mortality according to TyG-BMI. TyG-BMI: ​triglyceride glucose-body mass index​​. (Q1：<297.3，Q2：297.3–337.4，Q3：337.4–433.1，Q4：>433.1）

**Figure S3 The potential non-linear relationship between TyG-BMI and 90-/180-d mortality in patients with ASCVD was evaluated using a restricted cubic spline analysis**

**
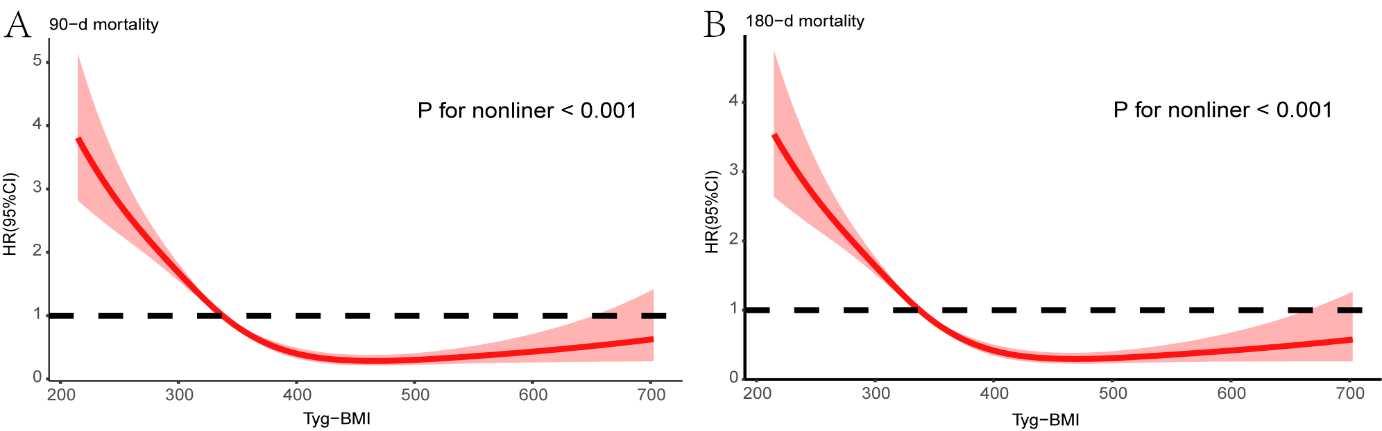
**

(A) A multiple-variable adjusted restricted cubic spline method was used to illustrate the relationship between TyG-BMI levels and 90-d mortality in ASCVD patients; (B) A multiple-variable adjusted restricted cubic spline method was used to illustrate the relationship between TyG-BMI levels and 180-d mortality in ASCVD patients. The shaded area represents the 95% confidence interval (CI). HR: hazard ratio; CI: confidence interval; TyG-BMI: ​triglyceride glucose-body mass index​​.

**Figure S4 ROC curve analysis of the incremental effect of TyG-BMI on 90-d all-cause mortality**

**
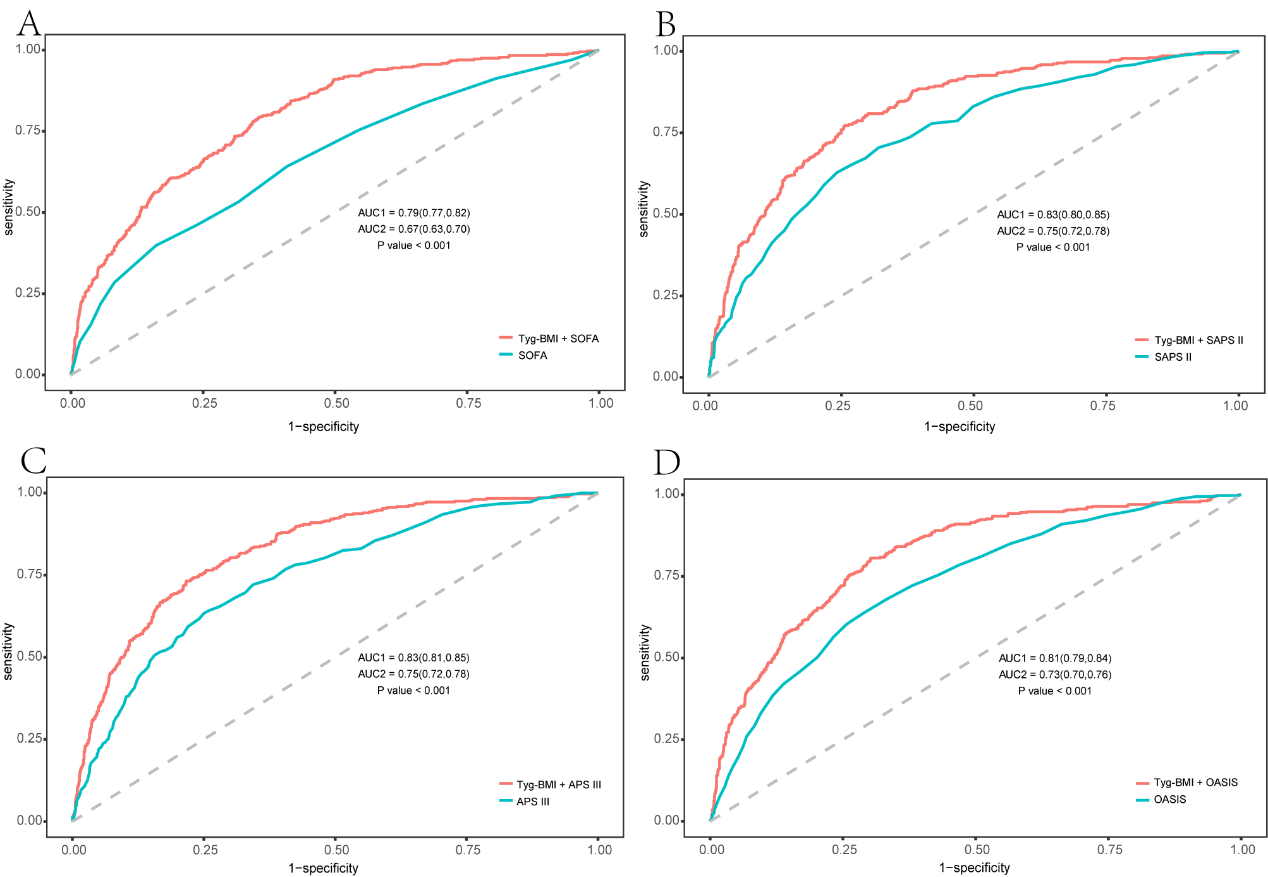
**

(A) SOFA + TyG-BMI; (B) SAPS II + TyG-BMI; (C) APS III + TyG-BMI; (D) OASIS + TyG-BMI. SOFA: Sequential Organ Failure Assessment; SAPS II: Simplified Acute Physiology Score II; APS III: Acute Physiology Score III; OASIS: Oxford Acute Severity of Illness Score; TyG-BMI: ​triglyceride glucose-body mass index​​.

**Figure S5 ROC curve analysis of the incremental effect of TyG-BMI on 180-d all-cause mortality**


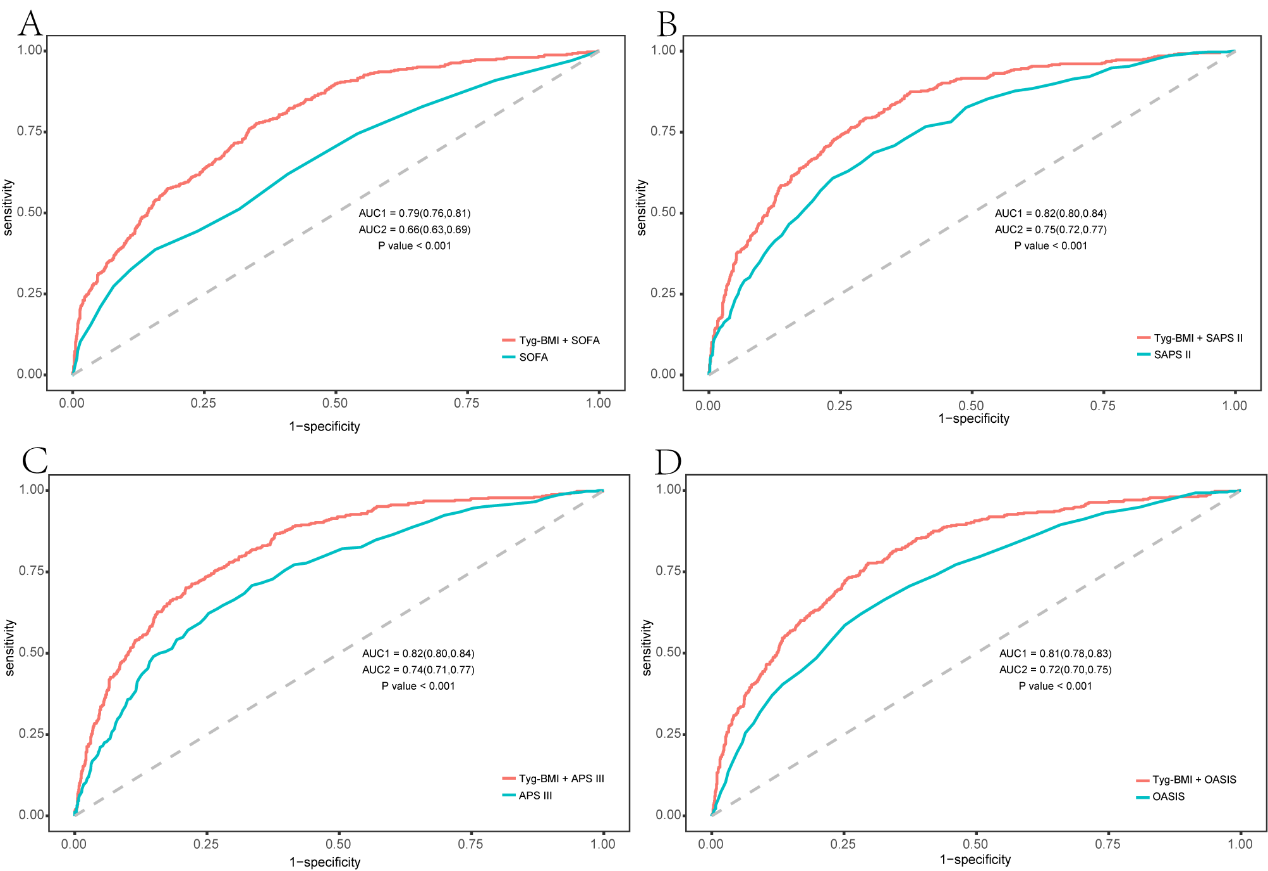


(A) SOFA + TyG-BMI; (B) SAPS II + TyG-BMI; (C) APS III + TyG-BMI; (D) OASIS + TyG-BMI. SOFA: Sequential Organ Failure Assessment; SAPS II: Simplified Acute Physiology Score II; APS III: Acute Physiology Score III; OASIS: Oxford Acute Severity of Illness Score; TyG-BMI: ​triglyceride glucose-body mass index​​.

**Figure S6 Subgroup analyses for the correlation of TyG-BMI with risk of 90-d and 180-d mortality in ASCVD patients**


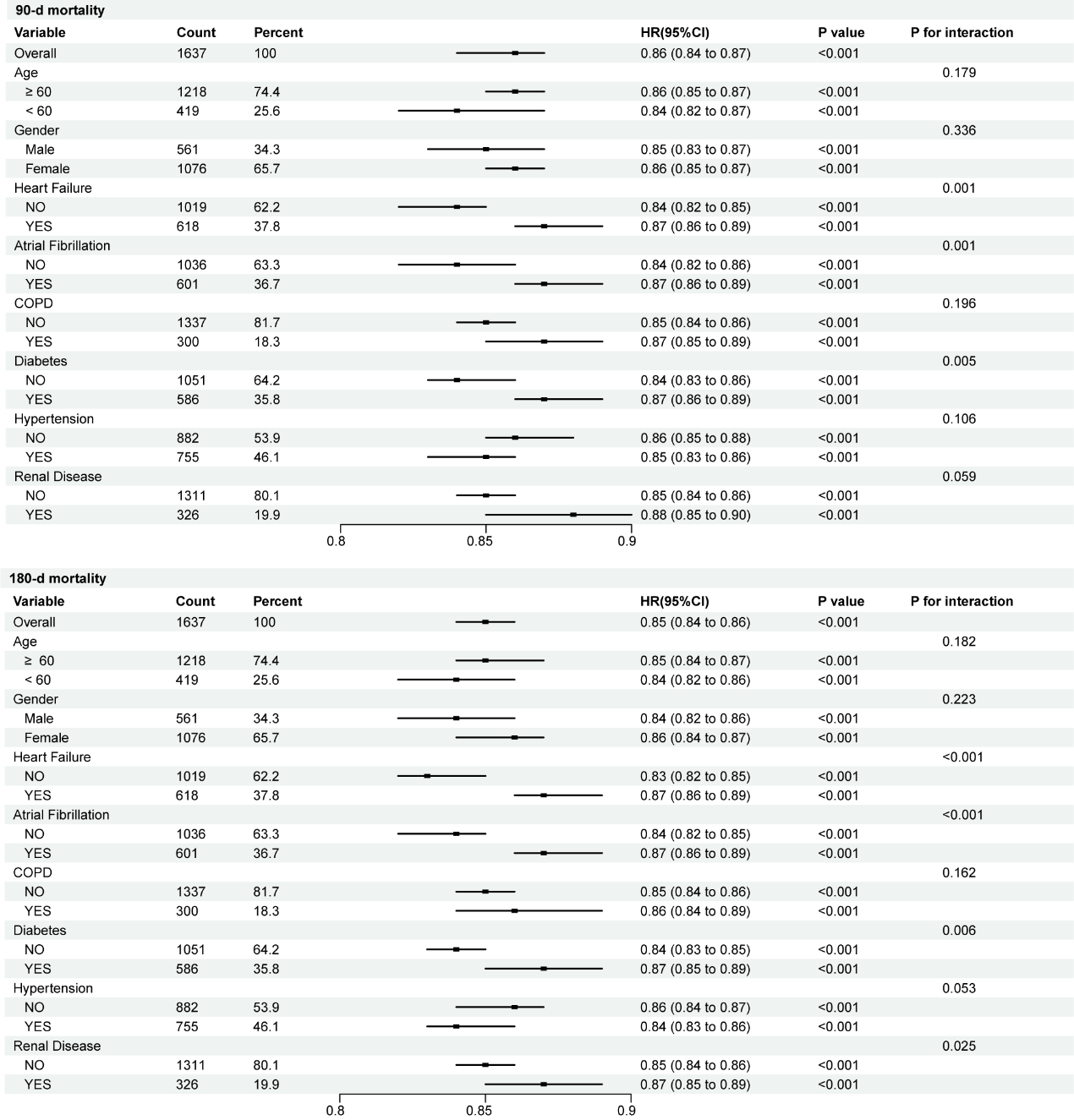


COPD: chronic obstructive pulmonary disease; HR: hazard ratio; CI: confidence interval.
